# *Humulus lupulus* L. in Animal Nutrition: Phytochemical Profile, Bioactive Properties, and Applications as a Functional Feed Additive—A Comprehensive Review

**DOI:** 10.3390/plants15111697

**Published:** 2026-05-30

**Authors:** Claudio Zepeda, Jéssica López, Carolina Figueroa, Constanza Low, Germán Olivares-Cantillano

**Affiliations:** 1 Escuela de Alimentos, Pontificia Universidad Católica de Valparaíso, Waddington 716, Playa Ancha, Valparaíso 2360100, Chile; claudio.zepeda.m@mail.pucv.cl; 2 Programa de Doctorado en Ciencias Agroalimentarias, Facultad de Ciencias Agronómicas y de los Alimentos, Pontificia Universidad Católica de Valparaíso, Valparaíso 2360100, Chile; 3 Escuela de Tecnología Médica, Pontificia Universidad Católica de Valparaíso, Avenida Universidad 330, Valparaíso 2360100, Chile; carolina.figueroa@pucv.cl; 4 Centro de Investigación de Acuicultura Sustentable (CIAS-PUCV), Escuela de Ciencias del Mar, Pontificia Universidad Católica de Valparaíso, Campus Curauma, Valparaíso 2360100, Chile; constanza.low@pucv.cl; 5 Piscicultura Río Blanco “Federico Albert Taupp”, Escuela de Ciencias del Mar, Pontificia Universidad Católica de Valparaíso, Valparaíso 2340000, Chile; german.olivares@pucv.cl

**Keywords:** hops, *Humulus lupulus*, xanthohumol, bitter acids, prenylated flavonoids, phytogenic additives, animal nutrition, antimicrobial activity, antioxidants

## Abstract

Hops (*Humulus lupulus* L.) are a phytochemical resource rich in bitter acids, prenylated flavonoids, and essential oils with antimicrobial, antifungal, antioxidant, and anti-inflammatory activities relevant to animal production. This review critically synthesizes the phytochemical profile of *H. lupulus* and the available in vivo evidence on its use as a functional feed additive in poultry, freshwater aquaculture, swine, and ruminants, identifying research gaps and regulatory barriers. In poultry, microencapsulated β-acids at 30 mg/kg feed achieved a feed conversion ratio comparable to zinc bacitracin, while lupulone reduced intestinal *Clostridium perfringens* counts by >4 log units, from log_10_ 6.20 to 2.00 CFU/g; doses ≥240 mg/kg induced adverse effects. In freshwater aquaculture, hop extract at 750 mg/kg feed improved hepatic markers and fillet fatty acid composition in common carp, whereas isolated hop acids at 308 mg/kg increased final body weight in Nile tilapia (157.3 vs. 150.3 g) without sensory rejection even at 1230 mg/kg. In swine, granulated cones improved feed conversion (3.5 vs. 4.3 kg/kg), while purified β-acids up to 360 mg/kg improved performance comparably to colistin. In ruminants, hop residues, pellets, and cones were tolerated without consistent production benefits. Overall, hop-derived additives show dose-, compound-, and matrix-dependent effects, requiring standardized formulations, species-specific pharmacokinetics, pathogen-challenge validation, long-term safety assessment, and regulatory dossiers.

## 1. Introduction

Hops (*Humulus lupulus*) stand out as a plant of global importance, traditionally used in the brewing industry for their contribution to the bitterness and aroma of the final product [1]. This climbing species of the *Cannabaceae* family has been valued for its female inflorescences, rich in secondary metabolites. However, over the past two decades, phytochemical research has revealed that these same compounds possess broad-spectrum pharmacological properties, including antimicrobial, anti-inflammatory, antioxidant, and antifungal activity, as demonstrated in both in vitro and animal models [2].

The secondary metabolites in hop cones are primarily composed of three major fractions, bitter acids (10–30% of dry weight (d.w.)), polyphenols (4–14% *w*/*w*), and essential oils (0.5–3.5% *w*/*w*), which collectively constitute a structurally diverse and biologically active group [1,3]. Among these, α-acids such as humulone and β-acids such as lupulone exhibit potent antimicrobial activity against Gram-positive bacteria by disrupting the membrane proton gradient, with demonstrated efficacy at low concentrations against pathogens of veterinary and food safety relevance [4]. Prenylated flavonoids, particularly xanthohumol and its derivatives isoxanthohumol and 8-prenylnaringenin, exert robust antioxidant and anti-inflammatory effects mediated by the modulation of key molecular pathways, including Nuclear factor kappa-light-chain-enhancer of activated B cells (NF-κB), Nuclear factor erythroid 2-related factor 2 (Nrf2), and Cyclooxygenase-2 (COX-2), and by their ability to neutralize reactive oxygen species (ROS) [5,6]. Additionally, the terpenoid fraction of the essential oil, dominated by β-myrcene, α-humulene, and β-caryophyllene, contributes antifungal, anti-inflammatory, and sedative properties through mechanisms distinct from those of the polyphenolic fraction, underscoring the functional complementarity of the phytochemical matrix of hops [1,3,7].

Despite this mechanistic evidence, the systematic use of *H. lupulus*-derived products as functional additives in animal production systems remains limited. In poultry, hops have been proposed as a phytogenic alternative to antibiotic growth promoters [8]. Nevertheless, the currently available body of evidence has four major limitations that restrict its translation into practical recommendations.

First, the studies exhibit marked methodological heterogeneity, associated with differences in the chemical form of the administered ingredient (whole cones, standardized extracts, or isolated compounds), the doses tested, the target species, and the response parameters analyzed. This heterogeneity is exacerbated by the limited analytical characterization of the active fraction. In the absence of harmonized quantification of α-acids, β-acids, and prenylated polyphenols, nominally equivalent doses are not biologically comparable across studies, which limits the construction of formal dose–response syntheses and the development of robust meta-analytic evaluations.

Second, the evidence is unevenly distributed across the main production sectors. Most controlled trials have been conducted in poultry, while research in swine farming is comparatively more recent. In aquatic organisms, the available information is restricted mainly to freshwater cyprinid fish, with no published nutritional studies in marine species, salmonids in the production phase, or penaeid crustaceans.

Third, ruminants constitute a special case within this landscape. Despite their global production relevance and the potential applications of hops in the selective modulation of ruminal fermentation, with direct implications for the mitigation of enteric methane and the improvement of nitrogen efficiency, in vivo evidence remains scarce. Furthermore, production results are heterogeneous, and current regulatory authorization for the use of commercial β-acid extract as an additive does not cover ruminant species.

Fourth, previous critical reviews have addressed the various production sectors in a fragmented manner, without systematically integrating an assessment of the methodological quality of primary studies or the analytical traceability of the formulations used. This limitation takes on particular importance in a context where the global livestock sector faces growing pressure to reduce antibiotic use, improve feed efficiency, and meet increasingly demanding standards for safety, sustainability, and production traceability.

In this context, the present review aims to provide a comprehensive and up-to-date analysis of the phytochemical profile of *Humulus lupulus* L., with particular emphasis on the biological activities most relevant to animal health and production, including antimicrobial, antifungal, antioxidant, anti-inflammatory, antiviral, and neuroprotective properties with potential relevance to animal welfare. In addition, this review critically evaluates the available in vivo evidence regarding its application as a functional additive in poultry, aquaculture, and swine farming, identifying the most significant research gaps that currently limit its implementation on a commercial scale.

## 2. Botanical Origin and Chemical Composition of *Humulus lupulus*

### 2.1. Phylogeny and Geographic Distribution

**Figure 1:**
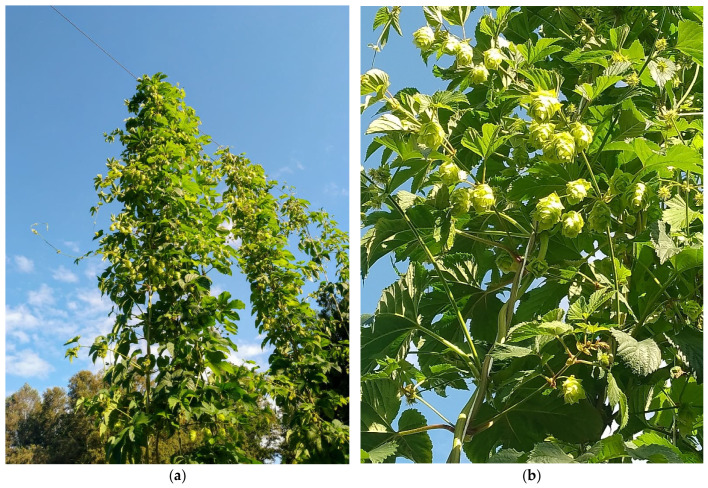
Representative views of *Humulus lupulus* plants and female cones. (**a**) Hop plants cultivated under intensive field production conditions. (**b**) Mature hop cones at harvest stage, corresponding to the plant structure used as the main source of bitter acids, prenylated flavonoids, and essential oils.

*Humulus lupulus* is a dioecious, perennial climbing plant belonging to the Cannabaceae family [1]. The characteristic morphology, from its cultivation in the field to the structure of the flower cones, is illustrated in Figure 1.

Phylogenetic analyses indicate that *Cannabis sativa* diverged approximately 27.8 million years ago [9,10]. This species exhibits optimal growth in temperate zones between latitudes 35 and 55° in both hemispheres, with commercial production mainly concentrated in Germany, the United States, China, and the Czech Republic [3]. Although approximately 97% of global hop production is destined for the brewing industry [3], recent research in phytochemistry has broadened interest in pharmaceutical and nutraceutical applications. This renewed focus is based on the complexity and richness of their chemical composition, which supports a diversity of bioactive properties with therapeutic potential [11].

### 2.2. Chemical Composition and Bioactive Compounds

**Figure 2:**
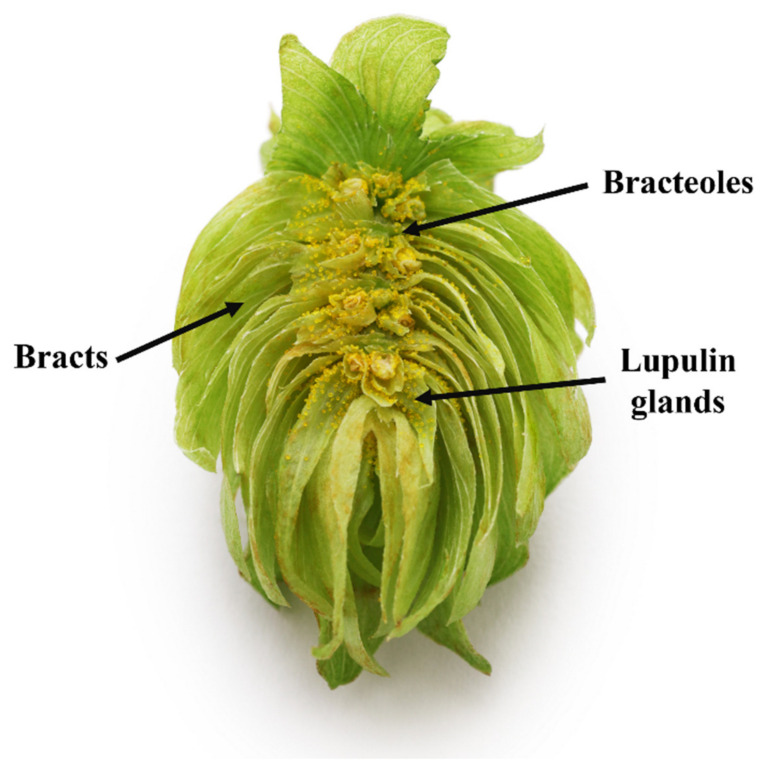
Cross-sectional structure of a typical hop cone. The image shows the main anatomical components of the cone, including bracts, bracteoles, and lupulin glands. Lupulin glands are specialized yellow secretory trichomes located mainly at the base of the bracteoles and are responsible for accumulating the principal bioactive metabolites of hops, including bitter acids, essential oils, and prenylated phenolic compounds.

Various studies have characterized the chemical composition of hops in detail, focusing on the female inflorescences, known as cones, and other plant structures. Once mature, the cones are covered with specialized trichome glands, also known as lupulin glands, visible as a distinctive yellow layer (Figure 2). These glands comprise a group of secretory cells that concentrate the main secondary metabolites of hops, including essential oils, resins, and prenylated phenolic compounds [12]. The average chemical composition of dry cones is detailed in Table 1. This structural diversity gives hops a wide range of antioxidant, antimicrobial, and pharmacological properties.

Although the cones concentrate the highest density of bioactive metabolites, the same compound classes, α- and β-acids, xanthohumol, and other prenylated flavonoids, also occur, at lower and variable concentrations, in vegetative structures such as the young hop shoots, which are produced in large amounts during the cultivation cycle and represent a complementary, largely underexploited source of hop phytochemicals [13]. Intraspecific variability is also substantial: in three Chilean hop ecotypes, total α-acids, β-acids, and xanthohumol showed marked intraspecific variation, with the Valdivia ecotype reaching 2.87% *w*/*w* α-acids, 6.49% *w*/*w* β-acids, and ~0.44% *w*/*w* xanthohumol on a dry weight basis (d.w.), underscoring the influence of genotype and growing region on the phytochemical profile [14].

**Table 1:** Average chemical composition of dried hop cones [2,3,15].

Compound	Amount (% *w*/*w* on Dry Basis)
Moisture	10
Proteins	15
Ash	8
Lipids	3
Sugars (Monosaccharides)	2
Amino acids	0.1
Essential oils	0.5–3
Resins	15–30
Polyphenols (tannins)	4
Pectins	2
Cellulose and Lignin	40
Waxes and steroids	traces–25
α-Acids (Bitter resins)	2–20
β-Acids (Bitter resins)	3–10

Essential oil accounts for approximately 0.5% to 3.5% of the dry weight of the trichome glands present in hop cones. Its composition is dominated by terpene hydrocarbons (monoterpenes such as β-myrcene and sesquiterpenes such as α-humulene), which constitute more than 90% of the total, while oxygenated compounds (such as linalool, geraniol, and farnesol) represent about 30%, contributing significantly to its aromatic profile and bioactivity [3,7]. β-Caryophyllene has garnered particular attention for its anti-inflammatory effects mediated by activation of the CB2 receptor (Cannabinoid Receptor Type 2) and for its ability to modulate the NF-κB pathway independently of the receptor—mechanisms relevant to applications in both human health and animal welfare [8]. Although sulphur compounds represent less than 1% of the total, they have low sensory thresholds and contribute significantly to aromatic notes, despite showing little biological activity [9].

Hop resins are a complex phytochemical fraction in which bitter acids predominate, representing between 10% and 30% of the dry weight of hops. Among these, α-acids (2–20% of dry weight) are dominated by humulone (35–70% of total α-acids), cohumulone (20–55% *w*/*w*), and adhumulone (10–15% *w*/*w*), while β-acids (3–10% *w*/*w*) are dominated by lupulone (30–55% of total β-acids). Additionally, hulupones, oxidation products of β-acids, are detected in proportions of 0.5% to 3.0% of dry weight [3,7,15].

The typical α:β ratio in commercial varieties ranges from 2:1 to 3:1. These compounds selectively inhibit Gram-positive bacteria by disrupting proton gradients and cation homeostasis. A recent meta-analysis that synthesized minimum inhibitory concentration (MIC) data from 18 studies reported weighted values of 12.40 µg/mL for lupulone and 21.92 µg/mL for xanthohumol against microorganisms of food and veterinary relevance [16]. Oxidation products of β-acids, such as hulupones (0.5–3.0% *w*/*w*), partially retain antimicrobial activity but are less chemically stable.

The recent expansion of the chemical space of β-acids deserves special mention. Zou et al. [17] used an activity-guided molecular networking approach to identify and characterize a series of new β-acid derivatives in hop cones with documented anti-inflammatory activity, demonstrating that the chemical diversity of this fraction remains incompletely characterized. Concurrently, Li et al. [18] isolated humupulones A and B—two acylfluoroglucin derivatives with a molecular skeleton previously uncharacterized in the genus *Humulus*—expanding the plant’s known phytochemical space with structurally novel compounds.

Prenylated flavonoids in hops—chalcones, flavanones, and flavones—constitute the most extensively studied phenolic class from a pharmacological perspective. Xanthohumol (XN, 2′,4′,4-trihydroxy-6′-methoxy-3′-prenylchalcone) is the major compound (0.1–1.0% dry weight) and acts as a biosynthetic precursor for other polyphenols of interest: isoxanthohumol (IXN) is formed by acid-catalyzed cyclization of XN, while 8-prenylnaringenin (8-PN)—the most potent known phytoestrogen—is generated by O-demethylation of IXN, both through intestinal microbial biotransformation and by chemical action under processing conditions [1,6]. These interconversions have direct implications for the compounds’ applications in biological systems: 8-PN exhibits significantly higher affinity for estrogen receptors than XN and IXN, resulting in a distinct activity profile and necessitating consideration of its formation in vivo when evaluating the bioactivity of XN-rich preparations. Optimizing extraction to maximize the recovery of prenylated flavonoids is a well-documented technological challenge: Haring et al. [19] demonstrated via accelerated solvent extraction (ASE) that XN and IXN exhibit opposite thermal sensitivities, and that 8-PN has a narrow temperature optimum, making it impossible to simultaneously maximize the recovery of all prenylated flavonoids under a single extraction condition.

The phytochemical composition of hops extends beyond the general bromatological categories outlined in Table 1 and encompasses a diverse array of bioactive compounds whose concentrations vary considerably among cultivars, ecotypes, growing regions, and post-harvest conditions. Table 2 presents the reported quantitative ranges for the principal groups of bioactive compounds, including bitter acids, prenylated flavonoids, essential oils, and other phenolic compounds, together with the primary sources of variability identified for each group.

Recent studies indicate that this variability arises from the interaction among genotype, whether cultivar or ecotype, geographic origin, and the edaphoclimatic conditions of the growing region [14,20,21,22,23,24]. Additional factors include harvest year, influenced by seasonal thermal stress [25], and harvest timing within a single season [26]. The quantitative implications are substantial, as the reported ranges for a given compound may differ by more than an order of magnitude depending on the material analyzed. For instance, the absolute concentration of β-myrcene has been reported to range from 1637 to 13,338 mg/kg, depending on cultivar, region, and harvest time [20,22,26].

Moreover, effective varietal discrimination and regional origin traceability require analytical approaches that extend beyond the quantification of total α- and β-acids. Essential oil composition, particularly the relative abundance of sesquiterpene markers such as β-farnesene, selinenes, and trans-α-bergamotene, provides additional chemotaxonomic resolution [27,28]. However, the practical implementation of these markers is constrained by the absence of established reference values for cultivars grown outside traditional production regions [29].

Collectively, these findings underscore that standardizing hops as a bioactive ingredient for animal production cannot rely on a single quantitative descriptor. Instead, it requires a multiparametric analytical strategy that explicitly accounts for the genotypic and geographic origin of the raw material. This compositional heterogeneity, in turn, may affect the consistency of the biological activities attributed to hops, which are discussed in the following section.

**Table 2:** Reported concentration ranges for the major bioactive compounds of *Humulus lupulus* and documented sources of intraspecific variability.

Compound Class/Compound	Reported Range (Unit)	Cultivar/Variety	Main Source of Variability	Reference
**Bitter acids**				
α-acids (total)	4.2–13.6 (% d.w.)	Cascade, Chinook	Cultivar × growing year	[21]
	0.48–3.97 (% sample weight)	Cultivars produced in Brazil	Cultivar × growing region	[22]
	3.8–7.9 (% *w*/*w*)	Cascade, Kazbek	Cultivar × geographic origin	[20]
	5.0–10.8 (% *w*/*w*)	Hallertau Mittelfrüher, Mapuche, Northern Brewer, Spalter, and Yakima Gold	Cultivar × growing season	[23]
	2.31–2.87 (% *w*/*w*)	Ranco, La Unión, Valdivia ecotypes	Ecotype genotype	[14]
	2.3–8.5 (% *w*/*w*)	Furano Beauty, Furano Magical, Cascade	Harvest timing	[26]
β-Acids (total)	2.4–6.2 (% d.w.)	Cascade	Cultivar × growing year	[21]
	0.40–1.51 (% sample weight)	Cultivars produced in Brazil	Cultivar × growing region	[22]
	3.6–7.3 (% *w*/*w*)	Cascade, Kazbek	Cultivar × geographic origin	[20]
	4.7–8.9 (% *w*/*w*)	Hallertau Mittelfrüher, Mapuche, Northern Brewer, Spalter, and Yakima Gold	Cultivar × growing season	[23]
	5.59–6.49 (% *w*/*w*)	Ranco, La Unión, Valdivia ecotypes	Ecotype genotype	[14]
		Furano Beauty, Furano Magical, Cascade	Harvest timing	[26]
Humulone	1.7–3.8 (% d.w.)	Callista	Harvest year	[25]
Lupulone	1.5–10.6 (% d.w.)	Callista	Harvest year	[25]
Cohumulone	0.4–0.9 (% d.w.)	Callista	Harvest year	[25]
	26.2–39.1 (% of total α-acids)	Cascade, Kazbek	Cultivar × geographic origin	[20]
Colupulone	0.9–6.5 (% d.w.)	Callista	Harvest year	[25]
	43.7–66.4 (% of total β-acids)	Cascade, Kazbek	Cultivar × geographic origin	[20]
**Prenylated flavonoids**				
Xanthohumol	0.01–0.3 (% sample weight)	Cultivars produced in Brazil	Cultivar × growing region	[22]
	0.49–6.01 (mg/g d.w.)	Varieties and Local Ecotypes Cultivated in Southern Chile	Genotype	[24]
	0.25–0.44 (% *w*/*w*)	Cascade, Kazbek	Cultivar × geographic origin	[20]
	0.34–0.44 (% *w*/*w* d.w.)	Ranco, La Unión, Valdivia ecotypes	Ecotype genotype	[14]
8-Prenylnaringenin	0.00–0.63 (mg/g d.w.)	Varieties and Local Ecotypes Cultivated in Southern Chile	Genotype	[24]
6-Prenylnaringenin	0.00–0.07 (mg/g d.w.)			
Desmethylxanthohumol	0.15–2.04 (mg/g d.w.)			
Isoxanthohumol	0.00–0.61 (mg/g d.w.)			
**Essential oil and terpenoids**				
Total essential oil	1.2–2.2 (mg/100 g)	Cultivars produced in Brazil	Cultivar × growing region	[22]
	0.91–1.03 (g/100 g)	Cascade, Kazbek	Cultivar × geographic origin	[20]
	0.6–1.3 (mg/100 g)	Hallertau Mittelfrüher, Mapuche, Northern Brewer, Spalter, and Yakima Gold	Cultivar × growing season	[23]
β-Myrcene	61.60–81.17 (% of essential oil)	Cultivars produced in Brazil	Cultivar × growing region	[22]
	1637–4606 (mg/kg)	Cascade, Kazbek	Cultivar × geographic origin	[20]
	5157–13,338 (mg/kg)	Furano Beauty, Furano Magical, Cascade	Harvest timing	[26]
Linalool	40.9–53.1 (mg/kg)	Cascade, Kazbek	Cultivar × geographic origin	[20]
	70.1–110 (mg/kg)	Furano Beauty, Furano Magical, Cascade	Harvest timing	[26]
Limonene	32.3–89.8 (mg/kg)	Furano Beauty, Furano Magical, Cascade	Harvest timing	[26]
α-Humulene	2.83–14.89 (% of essential oil)	Cultivars produced in Brazil	Cultivar × growing region	[22]
	70.7–2422 (mg/kg)	Furano Beauty, Furano Magical, Cascade	Harvest timing	[26]
β-Caryophyllene	0.18–18.52 (% of essential oil)	Cultivars produced in Brazil	Cultivar × growing region	[22]
	49.1–137 (mg/kg)	Furano Beauty, Furano Magical, Cascade	Harvest timing	[26]
β-Farnesene	19.0–710 (mg/kg)	Cascade, Kazbek	Cultivar × geographic origin	[20]
	491–1479 (mg/kg)	Furano Beauty, Furano Magical, Cascade	Harvest timing	[26]
Geranyl acetate	104–168 (mg/kg)	Cascade, Kazbek	Cultivar × geographic origin	[20]
Geranyl isobutyrate	88.4–125 (mg/kg)	Cascade, Kazbek	Cultivar × geographic origin	[20]
Geraniol	14.3–83.6 (mg/kg)	Furano Beauty, Furano Magical, Cascade	Harvest timing	[26]
**Phenolic acid and flavonoids**				
Gallic acid	0.18–0.52 (mg/g d.w.)	Varieties and Local Ecotypes Cultivated in Southern Chile	Genotype	[24]
	1.58–7.55 (mg/100 g)	Marynza, Lubelski, Magnum	Maturity level	[30]
Protocatechuic Acid	0.25–0.63 (mg/g d.w.)	Varieties and Local Ecotypes Cultivated in Southern Chile	Genotype	[24]
	1.07–5.58 (mg/100 g)	Marynza, Lubelski, Magnum	Maturity level	[30]
Catechin	0.31–0.83 (mg/g d.w.)	Varieties and Local Ecotypes Cultivated in Southern Chile	Genotype	[24]
Caffeic Acid	0.16–0.47 (mg/g d.w.)	Varieties and Local Ecotypes Cultivated in Southern Chile	Genotype	[24]
	4.23–42.9 (mg/100 g)	Marynza, Lubelski, Magnum	Maturity level	[30]
ρ-Coumaric Acid	0.05–0.22 (mg/g d.w.)	Varieties and Local Ecotypes Cultivated in Southern Chile	Genotype	[24]
	2.32–7.53 (mg/100 g)	Marynza, Lubelski, Magnum	Maturity level	[30]
Rutin	15.7–209.5 (mg/100 g)			
Kaempferol-3-glu	7.22–103.1 (mg/100 g)			

d.w.: dry weight; %*w*/*w*: weight to weight.

## 3. Pharmacological and Biological Properties of *Humulus lupulus*

The following section examines the main bioactive properties of hop and their relevance to human and animal health. The secondary metabolites of hops—bitter acids (α-acids such as humulone and β-acids such as lupulone), their isomerized and reduced forms, prenylated polyphenols (e.g., xanthohumol and isoxanthohumol), and essential oils—exhibit a broad spectrum of pharmacological activities documented across in vitro systems, mammalian models, and production animal studies, supporting their potential as multifunctional bioactive compounds with applications in both human health and animal production.

### 3.1. Antimicrobial Activity

The bioactive compounds in hop, particularly bitter acids such as humulone and lupulone, together with prenylated polyphenols such as XN, have demonstrated potent antimicrobial activity, especially against Gram-positive bacteria and some protozoa [31]. The Gram-positive selectivity of bitter acids determines their documented spectrum of activity against *Staphylococcus aureus*, *Listeria monocytogenes*, *Bacillus cereus*, *Clostridium perfringens*, *and Clostridium difficile*, among other foodborne and veterinary pathogens [4]. Their action is less effective against Gram-negative bacteria and fungi, due to structural differences in the cell envelope. The mechanism of action involves disruption of the proton gradient across the bacterial cell membrane via an ionophore-like process. In their lipophilic and protonated forms, these compounds traverse the lipid bilayer, dissociate within the alkaline cytoplasm, and release protons, thereby acidifying the intracellular environment [32].

Recent studies have shown that extracts of *Humulus lupulus* exert selective antimicrobial effects against pathogenic intestinal bacteria and, at the same time, promote a beneficial microbial composition [16].

In poultry models, lupulone has demonstrated efficacy against *Clostridium perfringens*, the etiological agent of necrotic enteritis in chickens [33]. Tillman et al. [34] observed that dietary supplementation with lupulone in broiler chickens resulted in the selective modulation of the gut microbiome, characterized by a reduction in pathogenic *Clostridium* spp. populations without affecting the abundance of beneficial bacteria such as *Lactobacillus* spp. This antimicrobial selectivity is particularly relevant in the design of feeding strategies that seek to preserve the homeostasis of the intestinal ecosystem without resorting to the use of broad-spectrum antibiotics. In another study involving broiler chickens, Bortoluzzi et al. [35] demonstrated that dietary supplementation with lupulone significantly reduced intestinal colonization by *Clostridium perfringens*, improved intestinal mucosal integrity, and decreased the incidence of enteritis.

Furthermore, some studies have reported that this antimicrobial activity not only reduces the pathogen load but can also indirectly modulate gut microbiota by preserving beneficial species, in contrast to broad-spectrum antibiotics [36].

### 3.2. Antifungal Activity

Dried strobiles (female flowers) and pellets of hops have demonstrated antifungal activity against zoosporic fungi, which are fungus-like organisms relevant to aquaculture, such as *Saprolegnia parasitica* and *Aphanomyces astaci*. These two species are responsible for severe infections in fish eggs and juveniles. In experimental trials with eggs *of Oncorhynchus tshawytscha* (Chinook salmon), treatment with these plant derivatives significantly delayed the appearance of fungal mycelium (the mass of fungal filaments) and reduced the progressive infestation of dead eggs [37]. In addition, a recent study by Nazımoğulları and Yanık [38] evaluated the efficacy of hop extracts as a natural disinfectant during the incubation of *Oncorhynchus mykiss* (rainbow trout) eggs. Daily applications of extract at concentrations of 0.25, 0.5, and 1 mg significantly reduced embryonic mortality and prevented the visible development of fungal colonization, even outperforming traditional commercial treatments. The authors attribute this effect to the action of hop bitter acids and suggest their use as an ecological strategy for the preventive control of saprolegniasis.

The antifungal activity of hops is related to their bitter resin fractions, particularly α- and β-acids, although the specific mechanisms against oomycetes have not yet been fully elucidated. It is postulated that these compounds may interfere with the integrity of the cell wall/membrane of fungi and oomycetes, affecting key enzymatic processes essential for mycelial growth [7,39].

### 3.3. Antioxidant Activity

The phenolic compounds present in hops, especially prenylated flavonoids such as xanthohumol, have demonstrated significant antioxidant activity both in vitro and in terrestrial animal models. This capacity manifests itself through various mechanisms, including free radical scavenging, transition metal chelation, and inhibition of pro-oxidant enzymes [1,7]. Beyond these direct mechanisms, xanthohumol (XN) also activates endogenous antioxidant defenses at the transcriptional level; XN-mediated nuclear translocation of nuclear factor erythroid 2-related factor 2 (Nrf2) induces the expression of phase II enzymes—NAD(P)H:quinone oxidoreductase-1 (NQO1), glutathione S-transferase, and heme oxygenase-1 (HO-1)—thereby enhancing cellular antioxidant capacity in a sustained and systemic manner [6].

Kłósek et al. [5], working with a hydroalcoholic extract of hop cones, were able to restore intracellular glutathione (GSH) levels and inhibit lipoperoxidation in infected cells, indicating a protective function against oxidative damage.

In vivo studies indicate that oral administration of hop extracts in mice significantly improves total antioxidant capacity, enhances the activity of enzymes such as superoxide dismutase (SOD), and reduces the levels of thiobarbituric acid reactive substances (TBARS) in the liver, thereby demonstrating efficacy against induced oxidative stress [40].

Alves et al. [41] further demonstrated that hop extract treatment in a murine model of obesity reversed glycemic imbalance and attenuated systemic oxidative stress, with significant increases in SOD activity and reduced brain degeneration mediated by modulation of Glycogen synthase kinase 3 beta (GSK3β) and phosphorylated insulin receptor substrate 1 (p-IRS1) proteins. Notably, Ortega et al. [42] showed that a complete hop extract, rather than isolated fractions, enhanced redox resilience and attenuated excitotoxic brain damage, suggesting synergistic interactions among the phytochemical matrix that favor the use of whole extracts over purified compounds in functional formulations.

In the context of animal production, Zawadzki et al. [43] demonstrated that dietary supplementation with hop β-acids at 30 mg/kg in broiler feed significantly improved meat redox stability, increasing the concentration of endogenous antioxidants such as carnosine, anserine, and reduced nicotinamide adenine dinucleotide (NADH), while reducing myofibrillar protein oxidation as evidenced by lower carbonyl group content.

### 3.4. Anti-Inflammatory Activity

The bioactive compounds in *Humulus lupulus*, particularly xanthohumol (XN), have demonstrated remarkable anti-inflammatory properties. This activity has been supported by various studies. For example, Cho et al. [44] studied the effect of XN on the production of inflammatory mediators, concluding that anti-inflammatory activity is mediated by the inhibition of key molecular pathways such as NF-κB, STAT-1α, and IRF-1, leading to a significant decrease in the expression of pro-inflammatory cytokines interleukin-1 beta (IL-1β), interleukin-6 (IL-6), tumor necrosis factor alpha (TNF-α) and pro-inflammatory enzymes such as inducible nitric oxide synthase (iNOS) and cyclooxygenase-2 (COX-2) in macrophages stimulated with lipopolysaccharide (LPS) and interferon gamma (IFN-γ). Bortoluzzi et al. [45] used an ex vivo model in pullets to show that hop β-acids can decrease LPS-induced IL-1β and INF-γ gene expression, confirming their direct anti-inflammatory effect on intestinal tissue. The researchers concluded that these hop compounds may help modulate the immune system in animals exposed to antigens.

Recent studies have shown that hop compounds, particularly prenylated derivatives such as prenylated chalcones, can modulate the inflammatory response in cellular models. Sangiovanni et al. [46] analyzed hydroalcoholic and aqueous extracts of hop cones (cv. Cascade) in a model of TNF-α-induced gastric inflammation and found that fraction D of the hydroalcoholic extract significantly reduced IL-8 secretion in human AGS human gastric adenocarcinoma epithelial cells. This inhibition, attributed mainly to prenylated chalcones, acted in part on the NF-κB signaling pathway.

In an in vitro and in vivo model of osteoarthritis (OA), Chen et al. [47] demonstrated that xanthohumol inhibits IL-1β-induced inflammation and extracellular matrix degradation in chondrocytes, preserves cartilage, and attenuates OA progression, likely through Nrf2/HO-1 activation and NF-κB inhibition. A comprehensive review by Kogut et al. [6] systematized the molecular targets of hop bioactives across NF-κB, nuclear factor erythroid 2-related factor 2 (Nrf2), adenosine monophosphate-activated protein kinase (AMPK), peroxisome proliferator-activated receptor (PPAR), and phosphoinositide 3-kinase/protein kinase B (PI3K/Akt) signaling pathways, confirming the multi-target anti-inflammatory profile of the hop phytochemical matrix. It should be noted, however, that these pathways have been characterized predominantly in non-productive mammalian systems, including rodents, human cell lines, and disease models such as osteoarthritis, hepatic steatosis, or neuroinflammation, so that their functional conservation and quantitative relevance in livestock and fish species remain a working hypothesis that requires species-specific experimental validation.

This interpretation fits within a broader context of plant-based additives with immunomodulatory properties. A particularly relevant example is hemp (*Cannabis sativa*), which, like hops, belongs to the Cannabaceae family. The synthesized evidence on dietary hemp and cannabinoids in animal production indicates that moderate levels of inclusion can improve immune status, health, and productive performance without adverse effects, while high levels can suppress immune function and reduce productive performance [48]. This biphasic, dose-dependent behavior, observed in a botanically related species, reinforces the notion that the benefits of phytogenic additives, including *H. lupulus*, depend on a defined dosage window and not on a linear dose–response relationship.

### 3.5. Antiviral Potential

The phytochemical profile of *Humulus lupulus* contains compounds with documented antiviral activity against clinically relevant enveloped and non-enveloped viruses, acting through at least three distinct mechanisms: destabilization of the viral envelope by the polyphenolic fraction [49], inhibition of viral replication by xanthohumol, demonstrated in vitro against the hepatitis C virus [50], and inhibition of the main coronavirus protease, including SARS-CoV-2 [51]. Furthermore, antiviral activity attributed to the prenylflavonoid fraction has been reported against enteroviruses and MERS-CoV [52]. The diversity of viral targets and their convergence with the previously described anti-inflammatory and antioxidant mechanisms suggest that the antiviral activity of hops could constitute a complementary property of its multifunctional matrix, rather than an isolated pharmacological activity.

However, the available evidence has been generated almost exclusively in viral models relevant to humans. To date, there are no published studies evaluating hop extracts or derivatives against viruses of production importance, including infectious bronchitis, Newcastle disease, and avian influenza in poultry; infectious pancreatic necrosis virus (IPNV) and infectious salmon anemia virus (ISAV) in salmon farming; and porcine reproductive and respiratory syndrome virus (PRRSV) in swine farming. Therefore, the relevance of the antiviral properties of hops for animal health and functional nutrition remains a working hypothesis. Its validation requires, first, in vitro assays in cell lines of the target species and, subsequently, in vivo studies under challenge with production-relevant viral pathogens.

### 3.6. Neuroprotective Properties

In addition to their antimicrobial, antioxidant, and anti-inflammatory effects, various compounds in *Humulus lupulus*, particularly prenylated flavonoids such as xanthohumol, modulate targets in the central nervous system, including oxidative stress, neuroinflammation, mitochondrial function, and GABAergic neurotransmission [6,53].

Although these activities have been studied primarily in rodent disease models and human cell lines [42,54,55], they are mechanistically relevant to a problem of growing economic importance in animal production: acute and chronic stress associated with handling, transport, high stocking density, and pre-slaughter handling. These factors can affect welfare indicators, immune competence, feed efficiency, and the oxidative quality of meat and fillets [48].

In this context, two mechanistic findings deserve special attention. First, xanthohumol-mediated activation of the Nrf2/HO-1 axis and inhibition of the NF-κB pathway, documented in multiple non-production models, converge with redox and inflammatory pathways also implicated in stress-induced oxidative damage in broiler chickens, finishing pigs, and farmed fish [6]. This convergence provides a biologically plausible basis for interpreting the antioxidant benefits observed in production trials with hop β-acids [43] and suggests that the neuroactive, antioxidant, and anti-inflammatory activities of the hop matrix may be functionally interrelated at the systemic level, rather than constituting independent properties.

Second, the phytochemical matrix of hops appears to act simultaneously on multiple complementary targets, an effect more consistent with the use of standardized whole extracts than with formulations based on isolated compounds [42]. This characteristic is operationally relevant for the development of functional additives, as it facilitates taking advantage of the complementarity among bitter acids, prenylated flavonoids, and terpenoids.

Despite this mechanistic plausibility, no in vivo studies are available that have directly evaluated the effect of hop-derived ingredients on stress-related welfare or performance indicators in poultry, swine, or aquaculture species. Therefore, the translation of these properties to production conditions remains a working hypothesis that requires species-specific validation. Future trials should consider controlled protocols involving stress from transport, handling, or stocking density, along with the simultaneous evaluation of cortisol or equivalent stress biomarkers, oxidative status, immune response, and production performance.

### 3.7. Sedating Effect

In the context of animal production, GABAergic modulation is an area of growing interest for managing acute stress associated with transport, handling, vaccination, and stocking density—situations in which conventional pharmacological sedation is not feasible due to regulatory, residue, and welfare restrictions.

The sedative properties of *Humulus lupulus* have been mainly associated with modulation of the GABAergic system, particularly through positive allosteric effects on GABA_A_ receptors mediated by bitter acids and prenylated flavonoids. In particular, humulone and 6-prenylnaringenin bind to sites distinct from the classical benzodiazepine site, with IC_50_ values of 3.2 and 3.7 µM, respectively, thereby potentiating GABAergic inhibitory tone [56]. At the extract level, Schiller et al. [57] demonstrated that ethanolic and supercritical CO_2_ extracts, administered orally at 100–200 mg/kg, significantly reduced spontaneous locomotor activity, prolonged ketamine-induced sleep, and lowered core body temperature in mice. These results support a central sedative effect associated primarily with the lipophilic fraction of hops. Complementarily, Brattström et al. [58] identified at least two pharmacologically distinguishable components within hop extracts: α-acids, particularly humulone, mainly associated with the sedative effect, and β-acids, linked to thermoregulatory effects.

However, it should be noted that these affinity parameters were determined in recombinant GABA_A_ receptors and rodent brain membranes. Since the subunit composition and pharmacology of GABA_A_ receptors may differ substantially among vertebrates, IC_50_ values and the magnitude of the sedative effect should not be directly extrapolated to fish or livestock species without specific functional validation. This caution is particularly relevant given the growing interest in the use of hops as a strategy for stress management in aquaculture.

A second consideration, rarely addressed explicitly in the available literature, is that the dose ranges associated with sedative effects in rodents, 100–200 mg/kg of extract, partially overlap with inclusion levels evaluated for production purposes in poultry, pigs, and freshwater fish, as discussed in Section 4. This raises the unresolved question of whether the productive effects of hop-based additives could be accompanied by subclinical sedative or motor effects that have not yet been systematically evaluated. Taken together, the sedative activity of *H. lupulus* appears to operate through multiple convergent mechanisms, including GABAergic receptor modulation and thermoregulatory effects, positioning it as a pharmacologically complex plant-derived sedative resource. However, the absence of controlled trials evaluating hop-derived ingredients as nutritional anti-stress agents in productive species, together with the lack of species-specific characterization of GABA_A_ receptor pharmacology in fish and livestock, remains a priority research gap before practical recommendations can be formulated.

Across the bioactivities reviewed, a recurring pattern emerges: the complete phytochemical matrix of hops appears to offer functional advantages over certain fractions or isolated compounds. Whole extracts have demonstrated greater redox resilience and neuroprotection than specific purified compounds in models of excitotoxicity [42], while the multi-target activation of signaling pathways such as NF-κB, Nrf2, AMPK, PPAR, and PI3K/Akt [6] is more plausibly explained by the concerted action of bitter acids, prenylated flavonoids, and terpenoids than by the activity of a single constituent. This functional complementarity, in which polyphenols primarily contribute to antioxidant and anti-inflammatory activity, bitter acids to antimicrobial action, and terpenoids to antifungal and sedative effects, supports the use of standardized whole matrices rather than formulations based on single compounds as a rational strategy for the development of functional additives.

However, translating this pharmacological promise into animal production and aquaculture faces specific barriers that the available evidence, mostly generated in in vitro models and in rodents, has not yet resolved. Among these are interspecies differences in gastrointestinal physiology, receptor pharmacology, and xenobiotic metabolism, which limit the direct extrapolation of effective concentrations; the limited oral bioavailability and extensive first-pass metabolism of prenylated flavonoids such as xanthohumol, whose tissue exposure at practical inclusion levels remains insufficiently characterized; interactions between the phytochemical matrix and food components, such as fiber, lipids, and proteins, which can modulate absorption, stability, and biological efficacy, as illustrated by the diet-dependent reversal of the antioxidant benefit in poultry diets enriched with polyunsaturated fatty acids [59]; and palatability constraints associated with the bitterness of α- and β-acids. Taken together, these challenges define the gap between mechanistic plausibility and application under production conditions, and justify the critical evaluation of the in vivo evidence presented in Section 4.

Although the pharmacological and biological properties have been characterized primarily in in vitro systems and laboratory animal models, a growing number of studies have begun to evaluate these bioactivities under production conditions in poultry, swine, freshwater fish, and ruminants, as discussed in detail in the following section.

## 4. Experimental Evidence of *Humulus lupulus* Supplementation in Production Animals

**Figure 3:**
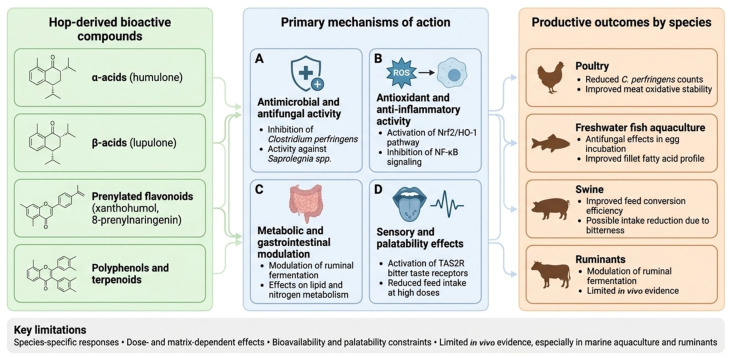
Schematic representation of the main mechanisms through which *Humulus lupulus* bioactive compounds may influence productive performance and health in farmed animal species. Hop-derived bioactive compounds, including α- and β-acids, prenylated flavonoids, polyphenols, and terpenoids, act through four major mechanisms: antimicrobial and antifungal activity (**A**), mainly associated with α- and β-acids and linked to the control of *Clostridium perfringens* in poultry and *Saprolegnia* spp. in freshwater fish; antioxidant and anti-inflammatory activity (**B**), primarily related to prenylated flavonoids and polyphenols and associated with improved meat oxidative stability and fillet quality; metabolic and gastrointestinal modulation (**C**), with potential relevance for feed conversion in swine and ruminal fermentation in ruminants; and sensory and palatability effects (**D**), largely associated with bitter compounds such as α- and β-acids, which may explain the reduction in feed intake observed at high inclusion levels, particularly in swine. The bottom panel summarizes the main modifiers and limitations of the available evidence, including dose, chemical form, delivery strategy, animal species, and the limited number of long-term in vivo studies. Abbreviations: NF-κB, nuclear factor kappa B; Nrf2/HO-1, nuclear factor erythroid 2-related factor 2/heme oxygenase-1; TAS2R, taste receptor type 2. Created with FigureLabs (figurelabs.ai).

The available in vivo evidence on the use of hop-derived bioactive compounds as functional additives in production animals is summarized in Table 3, Table 4, Table 5 and Table 6 and schematically integrated in Figure 3. These studies encompass poultry, swine, freshwater aquaculture, and ruminants, and collectively suggest dose-dependent, species-specific, and compound-specific effects on productive performance, intestinal and ruminal health, antioxidant status, and meat quality. However, the depth of evidence remains uneven across production systems. Poultry represents the most extensively studied model, with trials mainly focused on anticlostridial activity and the replacement of antibiotic growth promoters, whereas research in ruminants has primarily addressed the modulation of ruminal fermentation, reflecting the distinct bioavailability and mode of action of hop bitter acids in pre-gastric fermenters.

### 4.1. Poultry: Antibiotic Alternatives and Meat Quality

Poultry farming is the animal production system with the most extensive body of experimental evidence regarding the use of bioactive compounds derived from hops as an alternative to antibiotic growth promoters. In this context, broiler chickens (*Gallus* gallus domesticus) are the most widely studied species, and the in vivo and ex vivo studies conducted to date are summarized in Table 3. Siragusa et al. [33] demonstrated that administering lupulone (β-acid) in drinking water at concentrations of 62.5, 125, and 250 ppm, from day 13 to day 22 post-hatching, reduced intestinal counts of *Clostridium perfringens* in the jejunum and cecum by more than four logarithmic units (from log_10_ 6.20 to 2.00 CFU/g), with no significant differences among the various doses evaluated and no detectable effects on the birds’ final body weight, although the evaluation of zootechnical parameters was not the primary objective of the study. However, several aspects of the experimental design limit the productive extrapolation of these findings. Specifically, the administration period was relatively short, spanning only days 13–22 post-hatching, and the bioactive compounds were delivered via drinking water rather than incorporated into the feed. In addition, zootechnical parameters were not considered primary endpoints and were not evaluated under pathogen-challenge conditions. Moreover, the absence of significant differences among the three concentrations tested precludes the identification of a minimum effective dose. These methodological constraints highlight the need for further studies assessing dietary incorporation, longer supplementation periods, and challenge models that more closely reflect commercial production conditions.

Additionally, Tillman et al. [34] quantified using real-time PCR the effect of lupulone at 125 mg/L on the broiler’s intestinal microbiota, reporting a significant reduction in the *C. perfringens* subgroup (Cluster I) in both the cecum and the midgut, without significant alterations in the total microbiota of either segment. However, a significant reduction in *Lactobacillus* was observed in the midgut, an effect that the authors themselves identified as a relevant limitation, given that this genus represents an ecological barrier against pathogen colonization. The authors note that this reduction could favor the proliferation of *Enterobacteriaceae*, including *Salmonella* spp. and *Escherichia coli*, although this interaction did not reach statistical significance, possibly due to the study’s small sample size. It is worth noting that in the blind study, although *Lactobacillus* did not show a statistically significant reduction, the numerical values indicated a downward trend that should not be interpreted as active preservation of this population. Taken together, these findings suggest that lupulone exerts selective, but not completely specific, anticlostridial activity, which represents a critical consideration for its evaluation as an alternative to growth-promoting antibiotics.

At the dietary level, Bortoluzzi et al. [35] evaluated the effect of microencapsulated β-acids at four inclusion levels (30, 60, 120, and 240 mg/kg) over 42 days, using zinc bacitracin (30 mg/kg) as a positive control, under disease challenge conditions with coccidiosis vaccination and diets containing ingredients of animal origin. At 21 days, all treatments improved the feed conversion ratio (FCR) compared with the negative control, although zinc bacitracin achieved the most favorable value and was statistically superior to the treatments with β-acids. At 42 days, the inclusion of 30 mg/kg of β-acids resulted in an FCR statistically equivalent to that of the antibiotic control, which represents the main productive argument in favor of this compound as an alternative. However, the 240 mg/kg dose significantly reduced body weight and weight gain at 21 days compared to the negative control, demonstrating a dose-dependent adverse effect that limits the compound’s safety margin. The morphology of jejunal villi was not significantly affected by any treatment, although the 30 mg/kg treatment showed the highest villus-to-crypt ratio. Likewise, jejunal and cecal samples were virtually negative for *Clostridium* spp. across all experimental groups, including the negative control. This finding should be interpreted with caution, as the near-complete absence of *Clostridium* in the unsupplemented control indicates that the experimental model did not impose sufficient challenge pressure to discriminate among treatments. Consequently, the study does not allow the low clostridial load to be conclusively attributed to the antimicrobial activity of β-acids, thereby limiting the reproducibility and practical extrapolation of this outcome to commercial conditions in which necrotic enteritis pressure is effectively present.

From a mechanistic perspective, Bortoluzzi et al. [45] used an ex vivo model to evaluate the effect of hop β-acids on cytokine gene expression in ileal tissue from 10-week-old pullets exposed to lipopolysaccharide (LPS) as an inflammatory inducer. The results showed that β-acids significantly reduced IL-1β expression at both evaluated concentrations (30 and 240 mg/kg), whereas the reduction in IFN-γ was significant only at the higher dose (240 mg/kg), indicating a partially dose-dependent effect. IL-6 expression was not altered by any treatment, and the anti-inflammatory cytokines IL-4 and IL-10 also showed no significant changes, suggesting that the mechanism of action of β-acids operates primarily by suppressing the pro-inflammatory response, without parallel potentiation of the anti-inflammatory pathway. However, the ex vivo nature of the model, the use of pullet tissue rather than broiler chickens, and the absence of in vivo validation limit the direct extrapolation of these results to commercial production conditions; therefore, they should be considered preliminary mechanistic evidence requiring confirmation in more representative biological models.

Regarding the impact on the composition and oxidative quality of poultry meat, Zawadzki et al. [43] used quantitative nuclear magnetic resonance (NMR) spectroscopy and electron paramagnetic resonance (EPR) to evaluate the effect of four levels of microencapsulated β-carboxylic acids (0, 30, 60, and 240 mg/kg) on the metabolic profile and redox stability of broiler breast meat. The 30 mg/kg dose produced the lowest rate of free radical formation, as determined by EPR spin trapping, associated with higher concentrations of the endogenous antioxidants carnosine and reduced nicotinamide adenine dinucleotide (NADH), as well as a significant enrichment in long-chain polyunsaturated fatty acids, including arachidonic acid (ARA, C20:4*n* − 6) and docosahexaenoic acid (DHA, C22:6*n* − 3). This differential metabolic profile, characteristic exclusively of the moderate dose, suggests that β-fatty acids at low concentrations optimize intestinal absorption of polyunsaturated fatty acids (PUFAs) and promote the activity of long-chain desaturases, with direct benefits for the nutritional value of the meat. Concurrently, the myofibrillar proteins of broilers supplemented with β-fatty acids showed lower accumulation of carbonyl groups in both fresh meat and after seven days of refrigerated storage, a period during which the control group exhibited the greatest extent of protein oxidation. It is important to note, however, that the 240 mg/kg dose showed a divergent metabolic profile, with increased branched-chain amino acids and alterations in energy metabolism that the authors themselves interpret as indicators of metabolic stress and possible antinutritional effects at high doses, reinforcing the existence of a narrow optimal dose range for this compound.

In sharp contrast to these results, Rezar et al. [59] evaluated the effect of ground whole hop cones at two inclusion levels (0.9 and 3.6 g/kg) in diets with a high PUFA content (7.5% flaxseed oil), a deliberately pro-oxidant model. Supplementation at 3.6 g/kg significantly reduced body weight gain between days 21 and 37 and increased plasma MDA concentration, an indicator of systemic lipid peroxidation, whereas the 0.9 g/kg dose did not produce a significant effect. The MDA concentration in fresh meat was significantly higher in broilers supplemented with 3.6 g of hop cones per kg of feed compared to the control group, although differences between groups did not reach statistical significance in stored meat, suggesting that the pro-oxidant effect of whole hops manifests predominantly in the in vivo redox state and in meat immediately post-slaughter, with a lesser differential impact during the product’s shelf life. Additionally, both supplemented groups exhibited significantly lower concentrations of γ-tocopherol in fresh and chilled meat, a finding suggesting possible interference by hop components with the absorption or tissue retention of vitamin E, which would exacerbate oxidative vulnerability in dietary contexts rich in polyunsaturated fatty acids (PUFAs). Despite the pro-oxidant effect on lipids, the 3.6 g/kg dose significantly reduced lymphocyte DNA fragmentation as measured by the comet assay, an effect the authors attribute to the antigenotoxic properties of hop polyphenols, which operate via a mechanism independent of β-acids and decoupled from the lipid pro-oxidant effect.

The comparison between the results of Zawadzki et al. [43] and Rezar et al. [59] clearly illustrates that the biological response to hop supplementation is not uniform but is conditioned by at least three interdependent factors: the chemical form of the administered compound (microencapsulated purified β-acids vs. whole cones with a complex matrix), the inclusion dose, and the lipid composition of the base diet. In diets with conventional PUFA content, purified β-acids at moderate doses exert protective effects on meat oxidative quality; in diets enriched with highly unsaturated oils, administration of the whole plant can reverse this benefit and generate systemic oxidative stress. This compound–diet interaction represents a critical consideration for the design of supplementation strategies using bioactive compounds from hops in commercial poultry production.

Beyond this compound–diet effect, Kober et al. [8] review these main studies and make two key points: using whole cones works as well as purified β-acids without the extra effort to separate them, suggesting the dosage range depends partly on the form of the ingredient, not just on how much β-acids there are. Also, the reported benefits for chicken meat quality are based only on instrumental methods, without sensory or consumer panel validation of flavor acceptability.

This dependence on the source is not unique to hops but is a trait shared by other plant-based additives. In broilers, various plant tannins improved serum antioxidant capacity, strengthened intestinal barrier integrity, and shifted the immune profile toward an anti-inflammatory pattern, although the magnitude of these effects varied markedly by botanical source rather than by chemical class [60]. Taken together, these parallels place the antioxidant and immunomodulatory effects of hop β-acids within a broader pattern of phytogenic additives, in which the identity and origin of the extract, rather than the isolated nominal dose, govern the biological response and its interaction with the dietary matrix. This reinforces the need for ingredient characterization and standardization, as argued throughout this review.

### 4.2. Freshwater Fish: Preliminary Evidence in Cyprinids, Tilapiines, and Salmonid Egg Incubation

The scope of the available in vivo evidence on *H. lupulus* in aquatic organisms requires prior clarification. To date, published nutritional trials have focused on freshwater fish, primarily rainbow trout (*Oncorhynchus mykiss*), Nile tilapia (*Oreochromis niloticus*), and common carp (*Cyprinus carpio*). The in vivo studies evaluating the effects of hop supplementation in these species are summarized in Table 4. In contrast, no published nutritional studies are available on growing salmonids, marine teleosts of aquaculture interest, or penaeid crustaceans. The available reports on salmonids are limited to non-nutritional topical applications, such as the use of hop extracts for antifungal control of fertilized eggs, and therefore do not facilitate direct extrapolations to feeding regimes during the production phase. Consequently, the conclusions of this section should be interpreted within the context of a still-limited evidence base and should not be extended to other aquaculture species, farming systems, or production stages without specific experimental validation.

**Table 4:** Summary of in vivo studies evaluating the effects of *H. lupulus* supplementation on growth performance, fillet quality, antioxidant status, and hepatoprotection in freshwater fish (*Oncorhynchus mykiss*, *Oreochromis niloticus*, *Cyprinus carpio*).

Species/Model	Extract/Compound	Dose & Duration	Parameters Evaluated	Main Findings	Production Relevance	Reference
*Oreochromis niloticus* (Nile tilapia)	Hop bitter acids (humulone + lupulone; α-, β-, and iso-α-acids), Hopsteiner	0, 59, 308, and 1230 mg/kg in feed; 56 days (8 weeks)	Growth performance (weight gain, FCR, VSI, HSI, CF, fillet yield), plasma lysozyme activity, fillet LAB color, fillet aroma (triangle test, raw and baked, *n* = 69 panelists)	Hop acids showed no adverse effects up to 1230 mg/kg; the intermediate dose modestly improved late-stage body weight, with no effects on overall performance, immunity, fillet yield, or aroma acceptability	Antibiotic-free tilapia production; fillet food safety confirmed at high hop acid levels; supports valorization of spent brewer’s yeast as a sustainable aquafeed ingredient	[61]
*Oncorhynchus mykiss* (rainbow trout, fertilized egg incubation)	Ethanolic hop extract, 1:20 plant:ethanol ratio	0.25, 0.50, and 1.00 mg/L; daily 20 min bath treatment throughout incubation (33–35 days)	Hatching efficiency (%), larval survival rate (%); compared against commercial formaldehyde disinfectant and untreated control	Hop extract at 1 mg/L was the optimal treatment, significantly improving hatching efficiency and larval survival compared with both the untreated control and commercial disinfectant, while preventing visible fungal growth in treated eggs	Saprolegniosis control in salmonid hatcheries; natural ecological alternative to formaldehyde-based chemical disinfectants	[38]
*Oreochromis niloticus* (Nile tilapia)	Hop bitter acids (humulone + lupulone, Hopsteiner); spent brewer’s yeast (SBY, 12% inclusion, cider-derived)	300 mg hop acids/kg feed (HA); 12% SBY; 12% SBY + 300 mg hop acids/kg (SBY + HA); 70 days (10 weeks)	Growth performance (weight gain, FCR, SGR, VSI, HSI, CF, fillet yield), fillet LAB color, fillet proximate composition (protein, fat, ash)	Hop acids improved tilapia growth, but this benefit was lost when combined with spent brewer’s yeast, likely due to yeast-related antinutritional factors; survival remained 100% and fillet composition was unaffected	Supports the valorization of spent brewer’s yeast as a sustainable aquafeed ingredient, while confirming hop acids as a growth-promoting additive in tilapia; further SBY processing is needed to overcome antinutritional effects and enable combined benefits	[62]
*Cyprinus carpio* (common carp)	Common hop extract, commercial	Duration 0, 750 y 1500 mg/kg in feed; 8 weeks	Growth performance (FBW, SGR, FCR), whole-body proximate composition, fillet fatty acid profile, plasma antioxidant status, blood biochemistry	Hop extract did not affect growth but improved selected nutritional and hepatic markers in common carp. The 0.75 g/kg dose enriched fillet PUFAs and increased GSH, whereas 1.5 g/kg increased whole-body protein but also elevated LDH, suggesting possible metabolic burden at the higher dose	Nutritional quality improvement: 0.75 g/kg optimal for PUFA enrichment and hepatoprotection; caution at 1.5 g/kg due to elevated LDH indicating potential hepatic stress	[63]

SBY: spent brewer’s yeast; HA: hop acids; FCR: feed conversion ratio; FBW: final body weight; HSI: hepatosomatic index; LAB color: lightness, redness, and yellowness (Hunter color space); LDH: lactate dehydrogenase; SGR: specific growth rate; PUFA: polyunsaturated fatty acid; CF: condition factor.

In the field of aquaculture pathogen control, Nazımoğulları & Yanık [38] evaluated the bath treatment with an ethanol extract of hops at three concentrations (0.25, 0.50, and 1.00 mg/L) during the incubation of fertilized rainbow trout eggs (*Oncorhynchus mykiss*; 33–35 days), compared to a commercial formaldehyde disinfectant and an untreated control. The results showed dose-dependent improvements in hatching efficiency and larval survival rate, with the highest concentration (1.00 mg/L) yielding the highest values for both parameters (90.33% and 90.11%, respectively), significantly outperforming both the untreated control (84.67% and 85.22%) and the commercial disinfectant (86.33% and 86.44%). None of the hop-treated groups showed visible fungal growth during incubation. However, the study did not include quantitative microbiological analyses of *Saprolegnia*-type organisms, so antifungal activity is inferred indirectly from the viability results, which represents a methodological limitation that must be considered when interpreting these findings. Nevertheless, these results position hop extracts as a viable ecological alternative to conventional chemical disinfectants for the control of saprolegniasis in salmonid hatcheries, particularly relevant in the context of the progressive restriction of formaldehyde use in aquaculture.

At the dietary level, Lee et al. [61] evaluated, in a 56-day trial, the effects of isolated hop acids (humulone and lupulone) at four inclusion levels (approximately 0, 59, 308, and 1230 mg/kg of feed) on the production performance and fillet quality of Nile tilapia (*Oreochromis niloticus*). None of the doses evaluated produced significant differences in overall performance indicators—total weight gain, feed conversion ratio (FCR; kg of feed consumed per kg of weight gain), condition factor, fillet yield, or hepatosomatic index—although the visceral index was significantly lower in the low-dose treatment (59 mg/kg) compared to the control. Analysis of weekly body weight revealed that fish receiving the medium dose (308 mg/kg) reached a significantly higher weight than the control in weeks 7 and 8, suggesting a possible delayed-onset growth-promoting effect that was not reflected in the overall trial indicators. Plasma lysozyme activity did not differ among treatments, a result attributed by the authors to the controlled, pathogen-free conditions of the trial rather than to an absence of an immunomodulatory effect of the compound, highlighting the need for pathogen challenge studies to evaluate this aspect. Sensory evaluation by a consumer panel detected no perceptible differences in the aroma of the raw or cooked fillet, even at the highest dose evaluated (1230 mg/kg), confirming the organoleptic acceptability of the ingredient at inclusion levels of practical relevance. Complementing these results, a subsequent trial by the same group [62] examined the isolated and combined effects of spent brewer’s yeast (SBY, obtained from cider fermentations to exclude residual hop acids) and hop acids at 308 mg/kg in Nile tilapia over 70 days. Hop acids significantly improved final weight compared to the control (157.3 vs. 150.3 g), while the inclusion of 12% SBY reduced production yield by approximately 11% compared to the basal diet, an effect attributed to the yeast’s high nucleic acid content and the low urate oxidase activity characteristic of this species. The combination of SBY with hop acids did not restore performance, indicating that the antinutritional factors in the yeast may antagonize the growth-promoting effect of hop bitter acids, a critical consideration for the design of aquaculture diets based on byproducts of the brewing industry.

Building on previous findings regarding production performance, Dadras et al. [63] evaluated the effects of commercial hop extract at doses of 750 and 1500 mg/kg administered over eight weeks on body composition, fillet fatty acid profile, and plasma biochemical markers in common carp (*Cyprinus carpio*). Neither dose significantly altered production performance, including final weight, specific growth rate (SGR), or feed conversion ratio (FCR). However, both doses significantly reduced the activity of the liver enzymes alanine aminotransferase (ALT) and alkaline phosphatase (ALP), an effect interpreted as hepatoprotective and attributed to the antioxidant and enzyme-inhibiting activity of hop flavonoids, particularly xanthohumol. Additionally, the 750 mg/kg dose significantly enriched the fillet’s PUFA profile, including DHA (C22:6*n* − 3), docosapentaenoic acid (DPA, C22:5*n* − 6), and α-linolenic acid (C18:3*n* − 3), and increased plasma reduced glutathione (GSH) concentrations. However, the 1500 mg/kg dose significantly elevated lactate dehydrogenase (LDH), a marker of tissue damage and subclinical metabolic stress, a finding that coexists with reductions in ALT and ALP and reflects dissociated hepatic responses depending on the marker evaluated. This apparent contradiction among liver biomarkers at the highest dose does not necessarily represent an inconsistent effect, but rather the simultaneous expression of polyphenol-mediated hepatoprotective mechanisms and cellular stress induced by the phytochemical load at supra-optimal concentrations, confirming the existence of a narrow dosage window whose precise delimitation requires additional dose–response studies in this species.

Taken together, the four available in vivo trials in freshwater fish reveal three patterns of practical relevance for the design of future studies. First, the biological response appears to depend critically on the type of hop-derived product and its mode of administration. Thus, the bath application of an ethanolic extract during egg incubation [38], the dietary inclusion of isolated bitter acids [61,62], and the dietary inclusion of a complete commercial extract [63], produced qualitatively different results: antifungal protection, moderate growth stimulation, and hepatoprotection along with lipid enrichment of the fillet, respectively. This suggests that functional activity does not depend solely on the presence of common bioactive classes, but also on the specific composition of the product, the route of exposure, and the physiological context of the fish.

Second, the available evidence points to a narrow dosage window. In Nile tilapia, the intermediate bitter acid dose, 308 mg/kg, outperformed the highest dose, 1230 mg/kg, in weekly weight gain. Similarly, in common carp, the lower dose of the commercial extract (750 mg/kg) enriched the PUFA profile and increased plasma reduced glutathione, whereas the higher dose (1500 mg/kg) was associated with an increase in lactate dehydrogenase, consistent with a possible subclinical metabolic stress response. Taken together, these results suggest a nonlinear dose–response relationship, in which increases beyond an optimal range could reduce functional benefits or induce undesirable physiological responses. Third, immunomodulatory assessment criteria have so far been examined only under pathogen-free conditions [61]. Therefore, the ability of hop bioactives to confer protection under real infection pressure, which represents one of the most relevant outcomes from a production health perspective, remains unevaluated in freshwater fish and constitutes a priority direction for future research.

Taken together, the available in vivo evidence in the freshwater fish species demonstrates that the bioactive compounds of *H. lupulus* exert multifunctional effects—including indirect control of fungal pathogens, selective improvement of production performance, enrichment of the fillet’s fatty acid profile, and antioxidant and hepatoprotective activity—results consistently dependent on dose, the chemical form of the compound, the dietary matrix, and the recipient species. However, the presence of ingredient interactions, narrow dosing windows, and methodological gaps in the available studies collectively underscore the need for species-specific dose–response optimization studies, including pathogen challenges and quantitative microbiological analyses, before their implementation on a commercial scale in aquaculture can be recommended.

### 4.3. Swine: Feed Efficiency and Meat Nutritional Value

In pigs (*Sus scrofa domesticus*), evidence regarding the dietary effects of hops is still limited, though preliminary findings suggest potential applications in productive performance and meat quality. The in vivo studies evaluating the effects of hop supplementation in this species are summarized in Table 5. Belkova & Rozkot [64] evaluated the inclusion of granulated dried hop cones at two production stages: 0.5% in weaned piglets (21 days post-weaning) and 0.8% in finishing pigs. Although supplementation did not significantly alter average daily gain in either phase, finishing pigs in the experimental group exhibited a notable reduction in voluntary feed intake (2.73 vs. 3.05 kg/animal/day), which resulted in a substantial improvement in feed conversion ratio. This improvement in FCR, however, should be interpreted with caution: in the absence of palatability assessments or sensory acceptance tests, it is not possible to discriminate between an effect mediated by enhanced metabolic efficiency and one driven by reduced voluntary intake due to the bitter and astringent properties characteristic of hop bioactive compounds.

**Table 5:** Summary of in vivo studies evaluating the effects of *H. lupulus* supplementation on growth performance, feed efficiency, nutrient digestibility, and meat quality in swine (*Sus scrofa domesticus*).

Species/Model	Extract/Compound	Dose & Duration	Parameters Evaluated	Main Findings	Production Relevance	Reference
*Sus scrofa domesticus* (Prestice black-pied breed; weaned piglets and finishing pigs)	Dried granulated hop cones (variety Žatecký červeňák G 90); rich in polyphenols, bitter acids and essential oils	Stage I (weaned piglets): 0.5% hops/kg feed, 21 days post-weaning; Stage II (finishing pigs, ~70–120 kg): 0.8% hops/kg feed until slaughter	Growth performance, feed intake and FCR, fecal and meat composition, fatty acid and amino acid profiles, lipid oxidation during frozen storage, and lipid health indices	Hop supplementation did not affect growth, but reduced feed intake and improved FCR in finishing pigs. It also improved meat nutritional quality by lowering C14:0, reducing AI and TI, increasing selected fatty acids and lysine, and showing a trend toward lower lipid oxidation.	Supports dried hop cones as a functional feed additive in finishing pigs, improving FCR and meat nutritional quality through favorable fatty acid shifts, lower lipid health indices, and moderate antioxidant benefits without compromising growth performance.	[64]
*Sus scrofa domesticus* (weaned pigs, 35 d nursery)	Hop β-acids (microencapsulated, 30% active)	0, 120, 240, 360 mg/kg feed vs. colistin 40 mg/kg; 35 days	Growth performance (body weight, weight gain, feed intake, feed efficiency), nutrient/energy digestibility, diarrhea, organ weights, intestinal histology, microbial diversity, in vitro bacterial sensitivity)	Hop β-acids improved growth, feed efficiency, and fat digestibility, with performance comparable to colistin, but showed limited antimicrobial activity and a dose-dependent effect on diarrhea, and in vitro activity was limited to *Staphylococcus aureus*.	Antibiotic alternative, β-acids up to 360 mg/kg improved weanling-pig growth efficiency comparably to colistin, likely via enhanced fat digestibility (proposed activation of the peroxisome proliferator-activated receptor α).	[65]
*Sus scrofa domesticus* (growing pigs, ~60 kg initial BW)	Herbal extract from hop; BHT as positive control	500 or 1000 mg/kg feed; restricted individual feeding until slaughter	Body weight gain; apparent nutrient digestibility; carcass and meat quality; meat fatty acid profile and oxidative/color stability; blood biochemical indices; liver and kidney weights	Hop extract decreased fat digestibility; the higher dose (1000 mg/kg) lowered protein deposition and mean body weight gain; both doses reduced liver and kidney weights. Meat oxidative and color stability improved and the fatty acid profile shifted favorably.	Contrasting evidence: at high inclusion levels the herbal hop extract may impair growth and nutrient utilization, while still improving meat oxidative stability. Highlights dose- and preparation-dependent responses and the need for caution in extrapolating positive β-acid results.	[66]

FCR: feed conversion ratio; FBW: final body weight; TI: thrombogenicity index; AI: atherogenic index; BHT: butylated hydroxytoluene.

This appetite-suppressing effect has been attributed to the bitter acids in hops and their higher crude fiber content, consistent with what has been reported in humans by Walker et al. [67]. Chemical analysis of the musculus longissimus lumborum et thoracis (MLLT) muscle revealed significant changes in the lipid profile; a significant decrease in myristic acid (C14:0), known for its hypercholesterolemic effect, and significant increases in C15:1*n* − 5 and C24:1*n* − 9. Although differences in total saturated fatty acids (SFA), monounsaturated fatty acids (MUFA), and polyunsaturated fatty acids (PUFA) did not reach statistical significance, the overall trend was favorable, reflected in lower atherogenic and thrombogenic indices in the supplemented group. Regarding oxidative stability, malondialdehyde (MDA) levels assessed using the thiobarbituric acid reactive substances (TBARS) index on days 1, 3, and 6 post-thawing were consistently lower in the experimental group, although the differences did not reach statistical significance, possibly due to the small sample size. Regarding the amino acid profile, the significant increase in lysine content in the muscle of the supplemented group stands out; lysine is a limiting amino acid in swine nutrition and of high relevance in human nutrition. The underlying mechanism has not been elucidated, although the authors propose modulation of the gut microbiota by bioactive compounds in hops as a plausible mechanism. Taken together, these findings suggest that hop supplementation in finishing pigs may represent a low-cost nutritional strategy to improve production efficiency and generate a meat product with higher nutritional value; however, future studies should address the effects on the composition and functionality of the porcine gut microbiota, establish dose–response relationships with standardized formulations of bioactive compounds, and evaluate the impact on the sensory quality and shelf life of the final product.

Consistent with this, Sbardella et al. [65] evaluated dietary hop β-acids (120, 240, and 360 mg/kg) versus colistin (40 mg/kg) in 200 weanling pigs over a 35-day nursery period. Increasing β-acid levels linearly improved body weight, average daily gain, gain-to-feed ratio, and ether-extract digestibility, with no differences relative to the antibiotic control and no effect on feed intake; a quadratic effect on diarrhea occurrence was observed, and no effects on organ weights, small-intestine histology, or microbial diversity were detected. In vitro, only *Staphylococcus aureus* was sensitive to the β-acids. The authors proposed peroxisome proliferator-activated receptor alpha (PPARα) activation as a mechanism linking hop β-acids to improved lipid utilization. Together with Belkova & Rozkot [64], this study indicates that the favorable effects of hop on feed efficiency in pigs are reproducible across both purified β-acids and whole granulated cones, although the two studies differ in ingredient form, dose range, and life stage, precluding a unified dose recommendation.

This heterogeneity is also evident in the study by Hanczakowska et al. [66], who evaluated the inclusion of a herbal hop extract at 500 or 1000 mg/kg of diet in 96 finishing pigs, with an initial live weight of approximately 60 kg, maintained on an individually restricted feeding regimen. Unlike previous studies, supplementation with hop extract reduced the apparent digestibility of fat, while the higher dose decreased protein deposition and resulted in lower daily weight gain compared to the lower dose. Additionally, both doses reduced liver weight, and the 500 mg/kg dose decreased kidney weight compared to the control groups. However, the extract improved the oxidative and color stability of the meat, reduced its cholesterol content, and favorably modified the fatty acid profile, although the higher dose was associated with lower sensory acceptability of the meat.

Taken together, the three available in vivo studies in pigs indicate that the direction and magnitude of the response are inconsistent and appear to depend critically on the hop fraction administered—whether isolated β-acids, whole cones, or extracts—the inclusion dose, the production stage, and the experimental design. Consequently, although the current evidence in pigs is no longer limited to a single study, it remains scarce and heterogeneous. Therefore, it does not yet allow for a unified conclusion regarding the effect of hop supplementation on feed conversion, nutrient digestibility, or lipid metabolism, nor does it allow for the formulation of a sufficiently defensible dosage recommendation.

The effects observed for hops in pigs are consistent with those reported for other phytogenic additives evaluated in this species. In finishing pigs, *Litsea cubeba* essential oil (250–1000 mg/kg) did not significantly affect growth; however, at the optimal dose of 250 mg/kg, it improved the feed conversion ratio and increased the apparent digestibility of protein, ash, and calcium. In addition, an increase in serum catalase activity was observed, with no relevant changes in fecal microbial diversity at the phylum or genus level [68]. This pattern, characterized by improvements in feed efficiency and antioxidant status, but without consistent effects on growth or a marked restructuring of the microbiota, is consistent with what was observed for granulated hop cones and hop β-acids. Taken together, these findings reinforce the hypothesis that the effects of phytogenic additives in pigs are primarily related to improved digestive efficiency and oxidative balance, rather than to a direct stimulation of growth, while also suggesting the existence of an optimal dosage window below the maximum concentrations evaluated.

### 4.4. Ruminants: Ruminal Fermentation and Productive Performance

The available in vivo evidence on the use of *Humulus lupulus* in ruminants is limited. Unlike monogastric species, ruminants undergo extensive pre-gastric transformation of dietary components by the ruminal microbiota [69], which can alter the stability, bioavailability, and biological activity of hop-derived compounds. This consideration is particularly relevant because ruminal fermentation and the production of volatile fatty acids depend closely on diet composition, and because hop-derived compounds can modulate ruminal fermentation in a substrate-dependent manner. The available in vivo studies, summarized in Table 6, have primarily evaluated dry hop residues, silage hop residues, hop pellets, and hop cones as dietary ingredients or supplements in sheep and cattle. The available in vivo studies on the use of *H. lupulus* in ruminants are summarized in Table 6, which includes trials conducted in sheep and cattle using dry hop residues, ensiled hop residues, hop pellets, and hop cones as dietary ingredients or supplements.

**Table 6:** Summary of in vivo studies evaluating the effects of *H. lupulus* supplementation on intake, productive performance, ruminal fermentation, and metabolic parameters in ruminants (sheep and beef cattle).

Species/Model	Extract/Compound	Dose & Duration	Parameters Evaluated	Main Findings	Production Relevance	Reference
Sheep, adult crossbred, in vivo crossover trial	Air-dried hop residues	15% replacement of mixed hay; 63 g/kg BW; two 3-week periods	Plasma acetate concentration, acetate pool size, acetate turnover rate, ruminal VFA, plasma glucose, NEFAs and lactate	Hop residues showed no adverse metabolic effects, with most plasma metabolites unchanged and only a tendency toward lower acetate concentration.	Supports the partial use of dried hop residues as an alternative forage ingredient in sheep diets, although further nutritional evaluation is required.	[70]
Sheep, adult crossbred, in vivo crossover trial	Ensiled hop residues	Replacement of round bale silage with ensiled hop residues in a mixed hay-based diet; 63 g/kg BW; two 3-week periods	Dry matter digestibility, nitrogen digestibility and retention, rumen pH, ruminal VFA, plasma acetate concentration and turnover rate, NEFAs, lactate, glucose and plasma urea nitrogen	Hop diet maintained dry matter digestibility, acetate turnover, and nitrogen retention, but reduced nitrogen digestibility and plasma acetate without affecting NEFAs, lactate, glucose, or plasma urea nitrogen.	Dry matter digestibility and acetate turnover rate were similar between diets; however, N digestibility was significantly lower and plasma acetate concentration was significantly lower for the Hop-diet compared with the Hay-diet. N retention did not differ between diets.	[71]
Feedlot cattle, British × Charolais steers	Hop pellets, cv. Teamaker^®^	Growing phase: 0, 119, 238 a mg/kg DM; finishing phase: 0, 238, 476 and 952 mg/kg DM; 55-day growing and 105-day finishing periods	Feed intake, average daily gain, feed efficiency, carcass traits, fatty acid composition of diaphragm tissue and fecal *Escherichia coli* shedding	Dietary hops did not affect feed intake, growth, feed efficiency, carcass characteristics, tissue fatty acid composition or fecal *E. coli* shedding. The highest dose showed a numerical 6% increase in average daily gain, but this was not statistically significant.	Showed that hop supplementation at the tested levels was safe under feedlot conditions, but did not produce consistent improvements in productive performance.	[72]
Growing beef bulls, Slovene autochthonous Cika breed	Hop cones, cv. Aurora	50 or 100 g/animal/day, equivalent to 6 or 11 g hop DM/kg diet DM; 60 days	DMI, average daily gain, feed-to-gain ratio, plasma glucose, NEFAs, BHBA, urea, ALT, AST and GGT	Hop cones did not affect DMI, average daily gain or feed-to-gain ratio. Plasma glucose increased in both hop treatments, NEFAs decreased at the highest dose, and ALT was lower after 60 days.	Suggests that hop cones may influence energy metabolism and selected blood biochemical indicators, but without clear improvement in growth performance.	[73]

BW: body weight; VF: volatile fatty acid; NEFAs: nonesterified fatty acids; DM: dry matter; DMI: DM intake; BHBA: β-hydroxy butyrate; ALT; alanine aminotransferase; AST: aspartate aminotransferase; GGT: and γ-glutamyl transferase.

In sheep, the available in vivo evidence comes primarily from two studies by Al-Mamun et al. [70,71], conducted on six adult ewes using a crossover design to evaluate the use of dry or silage hop waste as a partial replacement for conventional forages. In the first study, 15% of a mixed-hay-based diet was replaced with dry hop residues during three-week experimental periods. Under these conditions, plasma acetate concentration tended to be lower in the hop-containing diet, while concentrations of glucose, non-esterified fatty acids, volatile fatty acids, and lactate did not differ between treatments. In the second study, silaged hop residues partially replaced conventional silage; dry matter digestibility and the plasma acetate turnover rate were similar between diets, although nitrogen digestibility was lower in the hop-containing diet, with no changes in nitrogen retention or in the major plasma metabolites. Taken together, these results indicate that hop residues, whether dry or ensiled, can be partially incorporated into sheep diets without apparent adverse metabolic effects. However, the evidence should be interpreted with caution, as both studies used a small number of animals, short experimental periods, and maintenance conditions, without evaluating production performance, palatability, detailed ruminal response, or feed efficiency. Therefore, these studies primarily support the feasibility of using hop residues as an alternative feed resource, rather than their efficacy as a functional additive capable of improving productivity in sheep.

In cattle, available in vivo evidence indicates relatively good tolerance to the inclusion of hops in the diet, but limited productive translation. Wang et al. [72] evaluated hop pellets in 60 British × Charolais steers fed diets based on barley silage and barley grain during the growth and finishing phases. Inclusion levels reached 476 mg/kg of dry matter in the growth diet and 952 mg/kg of dry matter in the finishing diet. Under these conditions, supplementation did not alter dry matter intake, daily weight gain, feed efficiency, carcass characteristics, fatty acid composition of diaphragm tissue, or fecal excretion of *Escherichia coli.* Although a numerical increase in daily weight gain was observed with the highest dose, this response did not reach statistical significance, limiting its interpretation as a real productive effect.

Similarly, Lavrenčič et al. evaluated hop cones in growing Cika bulls, using 50 or 100 g per animal per day, equivalent to 6 and 11 g of hop dry matter/kg of dietary dry matter. Although these doses provided higher concentrations of β-acids than those evaluated in some previous studies, supplementation did not affect dry matter intake, daily weight gain, or feed conversion. This result is relevant because it confirms that even relatively high levels of hop cones do not necessarily improve productive performance in cattle, despite the mechanistic plausibility derived from their ionophore-type antimicrobial activity. The authors observed, however, biochemical changes consistent with a modulation of energy metabolism: plasma glucose increased in the supplemented groups, serum NEFAs concentration decreased with the highest dose, and ALT activity was lower after 60 days of supplementation. These effects suggest that hop compounds can modify certain metabolic indicators in cattle, although without translating into measurable performance improvements. Overall, studies in cattle indicate that hops can be incorporated without evident adverse effects on intake, growth, or carcass yield, but their efficacy as a functional additive to improve feedlot performance remains weak. The discrepancy between the favorable effects observed in vitro on ruminal fermentation and the absence of consistent performance responses in vivo could be explained by the degradation or loss of activity of β-acids during processing, adaptation of the ruminal microbiota, the effective dose reaching the rumen, and the composition of the basal diet-factors that have been identified as limiting factors in studies of cattle supplemented with hops [36,72,74].

Taken together, the available in vivo studies indicate that hop residues, pellets, and cones can be partially incorporated into sheep and cattle diets without significant adverse effects on feed intake, growth, feed efficiency, carcass characteristics, or basic metabolic indicators. However, current evidence does not yet support a consistent productive response. In sheep, the main body of evidence concerns the feasibility of using dry or ensiled hop residues as alternative feed resources, though with potential effects on acetate metabolism and nitrogen digestibility. In cattle, supplementation with hop pellets or cones appears to be tolerated at different inclusion levels, but its effects are primarily expressed as metabolic modulation, particularly regarding glucose, non-esterified fatty acids (NEFAs), and liver enzymes, rather than as direct improvements in growth or feed conversion.

Although Table 6 includes only in vivo studies, in vitro evidence provides mechanistic support for the potential use of hops in ruminant nutrition. Hop-derived compounds can influence ruminal fermentation, the profile of volatile fatty acids, hyper-ammonia-producing bacteria, and methanogenic archaea. For example, Blaxland et al. [75] demonstrated that aqueous and commercial hop extracts reduced methane production by *Methanobrevibacter ruminantium* over a 30-day period, with commercial extracts of β-acids and tetrahydro-iso-α-acids being more persistent than aqueous extracts. However, the same study showed that the inhibitory effect varied among hop varieties and decreased over time in some aqueous extracts, reinforcing the need for phytochemical standardization and evaluation of response persistence.

Recent reviews on medicinal plants in ruminant nutrition support this broader interpretation. Plant-derived bioactive compounds can reduce enteric methanogenesis by modifying ruminal fermentation, redirecting metabolic hydrogen toward propionogenesis, and altering populations of methanogenic archaea and protozoa. However, their practical application is limited by variability in phytochemical composition, palatability, extraction costs, and the need for long-term in vivo validation. These limitations directly apply to hop-derived ingredients, whose bioactivity depends on the relative abundance of α-acids, β-acids, prenylated flavonoids, and other secondary metabolites [69].

Overall, the potential of *H. lupulus* in ruminants appears to be more closely related to the modulation of ruminal and metabolic processes than to a direct improvement in growth or feed efficiency. The gap between the promising effects observed in vitro and the limited production responses observed in vivo constitutes one of the main constraints to its practical application. Discrepancies between studies likely stem from differences in animal species, production stage, basal diet, method of administration, inclusion level, duration of supplementation, and actual concentration of α- and β-acids in the material used. Therefore, before recommending hop-derived ingredients as functional additives for ruminants, in vivo dose–response studies are required using chemically standardized formulations that simultaneously evaluate palatability, nitrogen utilization, ruminal fermentation, methane emissions, metabolic biomarkers, and long-term production performance.

Taken together, the available from poultry, aquaculture, swine, and ruminant production systems demonstrates that the bioactive compounds of *Humulus lupulus* L. exert multifunctional and species-specific effects on animal health, production efficiency, and the quality of the final product. The strongest body of evidence comes from poultry farming, where β-acids have demonstrated consistent anticlostridial and anti-inflammatory activity within well-defined dosage windows, with documented benefits for meat quality at moderate inclusion levels. In freshwater fish species to date (rainbow trout, Nile tilapia, common carp), available data supports the antifungal, growth-promoting, and hepatoprotective potential of hop acids and extracts, although results remain preliminary and require validation under commercial conditions and in pathogen challenge models. In swine, three independent studies evaluating distinct ingredient forms, granulated whole cones, purified β-acids, and herbal extracts, collectively support a favorable effect on feed conversion efficiency and on the lipid and amino acid profile of meat, although the magnitude and direction of the response vary substantially across studies, precluding a unified dose recommendation. In ruminants, the available in vivo evidence indicates that dried or ensiled hop residues, hop pellets, and hop cones can be incorporated into sheep and cattle diets without major adverse effects on feed intake, growth, carcass traits, or basic metabolic indicators; however, their productive benefits remain limited, and their main potential appears to be associated with modulation of ruminal fermentation, nitrogen utilization, and systemic energy metabolism.

Across the four production systems evaluated, a consistent pattern of dose-dependent and matrix-dependent biological effects emerges. This underscores that the translational potential of hop-derived bioactives can only be fully realized through rigorous optimization strategies tailored to each animal species, production stage, ingredient matrix, and target bioactive fraction.

## 5. Research Gaps, Regulatory Considerations, and Future Directions

Despite the growing body of evidence supporting the multifunctional potential of bioactive compounds from *Humulus lupulus* L. in animal production, the translation of this evidence into commercial applications across poultry, swine, freshwater aquaculture, and ruminant systems is constrained by critical knowledge gaps that must be addressed prior to large-scale implementation. The following subsections delineate these priorities in a logical sequence: first, the pharmacokinetic and metabolic characterization required to underpin rational dose design (Section 5.1); second, the optimization of dose–response relationships and formulation standardization (Section 5.2); third, the validation of efficacy under pathogen challenge models relevant to each production system (Section 5.3); fourth, the characterization of gut microbiota modulation as a central mechanistic axis (Section 5.4); fifth, the assessment of long-term safety, residue dynamics, and regulatory framework alignment (Section 5.5); and sixth, the resolution of palatability and feed intake barriers inherent to the bitter profile of hop α- and β-acids (Section 5.6). Together, these axes define the research agenda that must precede the consolidation of *H. lupulus* as a functional additive in commercial animal nutrition.

### 5.1. Pharmacokinetic and Metabolic Characterization

The bioavailability, tissue distribution, and metabolism of the bioactive compounds in hops, particularly α- and β-acids, xanthohumol, and their prenylated derivatives, have not been systematically characterized in the major food-producing species, including broiler chickens, pigs, Atlantic salmon, Nile tilapia, and cattle. The available pharmacokinetic information comes predominantly from rodent and human models in pharmacological or nutraceutical contexts; therefore, its extrapolation to production animals remains limited. This limitation reflects substantial species-specific differences in gastrointestinal pH, bile salt composition, intestinal transit time, the structure and activity of microbial communities, and hepatic enzyme profiles involved in xenobiotic metabolism. In the absence of species-specific pharmacokinetic data, rational dose design remains constrained, and withdrawal periods that ensure the safety of animal-derived products intended for human consumption cannot be established.

Several aspects warrant special attention. First, xanthohumol may undergo rapid isomerization to isoxanthohumol during thermal feed processing and within the gastrointestinal tract. Furthermore, it exhibits limited oral bioavailability in non-production models, primarily due to extensive phase II conjugation, including glucuronidation and sulfation. However, whether these metabolic pathways operate with comparable efficiency in poultry, swine, or aquaculture species remains unknown. Second, in ruminants, ruminal microbial biotransformation may inactivate or structurally modify α- and β-acids before intestinal absorption. This phenomenon may help explain the limited productive responses reported in feedlot cattle, in contrast to the marked anticlostridial effects observed in monogastric species (Section 4.4). Third, in farmed fish, pharmacokinetic behavior is further influenced by poikilothermy, which modulates intestinal absorption and hepatic biotransformation rates, as well as by gill excretion pathways that have no direct equivalent in terrestrial species.

To address this gap, species-specific pharmacokinetic studies are required, including the administration of single and multiple doses through routes applicable under production conditions, such as feed or drinking water. These studies should quantify parent compounds and their major metabolites in plasma, edible tissues, muscle, liver, and fat, and excretion matrices, such as eggs or milk where applicable, using validated analytical methods based on liquid chromatography–tandem mass spectrometry (LC–MS/MS). Likewise, key pharmacokinetic parameters should be determined, including maximum plasma concentration (C_max_), time to reach maximum concentration (T_max_), area under the plasma concentration–time curve (AUC), elimination half-life (t½), and tissue depletion curves. The absence of this baseline information represents one of the most significant knowledge gaps, as it constrains the interpretation of dose–response, efficacy, and safety studies analyzed in the following subsections.

### 5.2. Dose–Response Optimization and Formulation Standardization

Available studies reveal dose-dependent biological effects, with apparently narrow margins between functional efficacy and potential adverse responses. For example, moderate doses of β-acids, such as 30 mg/kg in poultry, have been associated with improvements in meat quality and indicators of production efficiency, while high doses of whole cones, such as 3.6 g/kg, have produced pro-oxidant responses and reduced performance, particularly in diets enriched with polyunsaturated fatty acids. Although the European Food Safety Authority concluded that a commercial extract of β-hop acids is safe as a sensory additive for pigs at a maximum inclusion rate of 50 mg/kg of complete feed (EFSA, 2018; Implementing Regulation (EU) 2019/111 [76]), no No Observed Adverse Effect Level (NOAEL) values have yet been published in the peer-reviewed literature for poultry, aquaculture species, or ruminants. Nor are there any comparative subchronic studies that directly evaluate the safety profile of hops, in their various forms, including whole cones, enriched extracts, and isolated fractions, against that of conventional feed components, such as soybean meal or other plant sources of polyphenols. The chronic, regulatory, and residue-related dimensions associated with this evidence gap are addressed in Section 5.5.

This interaction between compound, diet, and dose reinforces the need for dose–response studies based on standardized and analytically characterized formulations. In this context, the quantification of α-acids, β-acids, and prenylated polyphenols should constitute a mandatory quality control parameter. The comparative interpretation of safety margins is further complicated by the wide intraspecific variability in hop phytochemistry across genotypes and growing regions, which can alter the effectively administered dose of bioactive compounds even when the dietary inclusion rate remains constant. This variability, however, also offers an opportunity for regional valorization. Locally adapted ecotypes, such as the Valdivia, Ranco, and La Unión accessions recently characterized in southern Chile, with distinct profiles of α-/β-acids and xanthohumol, could serve as standardized sources of bioactive material, provided their composition is verified by HPLC or LC–MS/MS prior to use as an additive [14].

Formulation strategies should also consider hop-derived materials beyond the cones. Hop shoots, produced in significant quantities during the production cycle and currently underutilized, retain the main classes of bioactive compounds, including α- and β-acids, xanthohumol, and other prenylated flavonoids, although in varying concentrations and, in general, lower than those observed in the cones. Their incorporation into animal feed could represent a complementary stream of byproducts, aligned with the principles of the circular bioeconomy. However, their use requires specific dose–response, palatability, and safety trials before commercial recommendations can be made [13]. In parallel, the development of stable delivery systems, such as microencapsulation, lipid carriers, and bitterness-masking strategies, is essential to improve palatability, protect bioactive compounds, and ensure ingredient stability in commercial feed matrices. These aspects are discussed in greater detail in Section 5.6, regarding palatability, and in Section 5.5, regarding their economic and regulatory implications.

### 5.3. Validation in Pathogen Challenge Models

The antimicrobial and antifungal activity of hop-derived bioactive compounds has been documented primarily under in vitro conditions or in pathogen-free experimental models, which limits the predictive value of the available evidence regarding their protective efficacy under actual infectious pressure. Therefore, validating this efficacy in controlled challenge models using pathogens relevant to each production system represents a critical step before recommending their commercial application.

Priority pathogens differ across production systems. In poultry, necrotic enteritis induced by *Clostridium perfringens* represents the most clinically and economically relevant challenge model, ideally complemented by co-infection protocols with *Eimeria* spp., which more accurately reproduce disease dynamics under field conditions. Likewise, *Salmonella Typhimurium* and *Campylobacter jejuni* constitute additional priority targets due to their dual zoonotic and productive relevance. In freshwater aquaculture, *Aeromonas hydrophila* and *Streptococcus agalactiae* in Nile tilapia, *Aeromonas hydrophila* and koi herpesvirus in common carp, and *Saprolegnia parasitica* in cyprinid and salmonid systems represent relevant targets, given that the available in vivo evidence on hops is primarily concentrated in these species. In swine, enterotoxigenic *Escherichia coli* (ETEC) in newly weaned piglets and *Lawsonia intracellularis* in growing–finishing pigs are priority models. In ruminants, *Fusobacterium necrophorum*, associated with liver abscesses in beef cattle, *Mannheimia haemolytica*, linked to bovine respiratory disease, and experimentally induced subacute ruminal acidosis, as a non-infectious challenge model, constitute appropriate evaluation frameworks. The latter is particularly relevant given the mechanistic plausibility that hop β-acids may modulate lactate-producing ruminal bacterial populations.

Challenge trials should employ designs with sufficient methodological rigor, including sample sizes determined through statistical power analysis, well-characterized challenge inocula with standardized infectious doses, positive controls using reference antibiotics or antifungals at therapeutic doses, and the concurrent evaluation of productive parameters, clinical signs, mortality, bacterial, fungal, or viral loads in target tissues, histopathological lesion scores, and molecular biomarkers of the host response. Among the latter, the expression of inflammatory cytokines, acute-phase proteins, tight junction proteins, and markers of antioxidant defense should be considered. The simultaneous integration of productive, microbiological, histopathological, and molecular variables would enable discrimination between direct antimicrobial effects and host-mediated responses, such as immunomodulation or mucosal barrier protection—a distinction that currently cannot be clearly established based on the available evidence.

### 5.4. Microbiota, Ecological Selectivity, and Host-Mediated Functional Responses

The selective antimicrobial activity of hop-derived bioactive compounds, with a documented preference for Gram-positive bacteria, raises important questions regarding their impact on the composition, diversity, and function of the gastrointestinal microbiota in food-producing species. The preservation of beneficial taxa, such as *Lactobacillus* spp., observed in some poultry studies is encouraging; however, the available evidence remains primarily limited to targeted bacterial counts and does not capture the full ecological consequences of dietary hop inclusion.

Several methodological priorities can be identified. First, the systematic application of high-throughput sequencing approaches is required, including taxonomic profiling based on the 16S rRNA gene, shotgun metagenomics for functional gene inference, and metatranscriptomics or metabolomics to assess active microbial functions. These tools would enable a higher-resolution characterization of the structural and functional changes induced by hop supplementation in each target species. Second, microbiota studies in production animals have traditionally focused on the bacterial component; however, a comprehensive assessment should also include the archaeal community, which is particularly relevant in ruminants and in the modulation of methanogenesis, as well as intestinal anaerobic fungi, protozoa, the virome, and the intestinal mycobiome—ecological compartments increasingly recognized as relevant components of host health.

Third, the structural and functional differences among the gastrointestinal ecosystems of the species analyzed require experimental designs specific to each production system. The pre-gastric fermentation chamber of ruminants, the monogastric gastrointestinal tract with relevant cecal fermentation in poultry, the segmented intestinal tract of pigs, and the comparatively short intestine with lower bacterial density in farmed fish represent fundamentally distinct microbial habitats. Therefore, results obtained in one system should not be extrapolated to another without specific validation.

Fourth, microbiota studies should be integrated with functional indicators of gut health, including short-chain fatty acid profiles; markers of intestinal barrier integrity, such as the expression of tight junction proteins, zonulin, or fecal calprotectin where applicable; indicators of mucosal immunity, such as secretory IgA, mucin-2 expression, and regulatory T-cell populations; and metabolomic profiling of the gut–liver axis. Only through this integrated approach will it be possible to adequately assess the potential of hop-derived bioactive compounds to favorably modulate the microbiota–immunity–metabolism axis in each target species, distinguishing transient compositional changes from sustained functional benefits.

### 5.5. Long-Term Safety, Residues, and Regulatory Framework

Most of the available studies are limited to short experimental periods (3–10 weeks), which are insufficient to assess effects on reproductive parameters, chronic liver and kidney function, or the dynamics of residues in edible tissues throughout the entire production cycle. The determination of maximum residue limits (MRLs) in muscle, liver, and fat using validated analytical methods, such as HPLC-MS/MS, is generally required for regulatory approval in most jurisdictions. In milk- and egg-producing species, this characterization must also extend to the potential transfer of hop-derived compounds and their metabolites into these matrices, an aspect that has not yet been investigated and constitutes a relevant additional regulatory requirement.

Based on the dose–response and acute safety considerations discussed in Section 5.2, the chronic, residue, and regulatory aspects present additional knowledge gaps. A specific safety consideration regarding *Humulus lupulus*, which distinguishes it from most phyto-additives, is the presence of 8-prenylnaringenin, recognized as one of the most potent phytoestrogens described to date, along with other prenylflavonoids with documented endocrine activity. The implications of chronic dietary exposure on the reproductive performance of breeding animals, as well as the potential transfer of these estrogenic metabolites to edible matrices such as milk and eggs, have not been sufficiently characterized and constitute critical aspects for regulatory evaluation prior to eventual commercial implementation. Furthermore, No Observed Adverse Effect Levels (NOAELs) and safety margins regarding effective inclusion rates for the target species have not been systematically established. This gap prevents a formal risk characterization comparable to that available for conventional feed ingredients.

The regulatory framework presents some favorable precedents worth considering. In the United States, hops (*Humulus lupulus* L.) and several of their essential oils, oleoresins, and natural extracts are listed as Generally Recognized As Safe (GRAS) for use in human food under 21 CFR §182.20 [77]. This precedent represents a comparative advantage over other phytogenic additives, as it provides a previously established history of use and safety. However, GRAS recognition for human food applications does not automatically imply approval as an additive in animal feed, which requires specific regulatory evaluations based on the species and intended use. In the European Union, certain hop derivatives are authorized as sensory additives (flavorings) in animal feed under Regulation (EC) No. 1831/2003. However, regulatory dossiers for aquaculture and swine must address additional considerations, including the assessment of the environmental impact of compounds excreted into aquatic effluents, the demonstration that hop compounds do not promote the selection of resistant bacterial populations, and compliance with Good Manufacturing Practices (GMP) during the production of the additive.

In addition, various prenylated flavonoids in hops, particularly xanthohumol, isoxanthohumol, and 8-prenylnaringenin, inhibit human cytochrome P450 (CYP450) enzymes in vitro, particularly the CYP2C and CYP1A2 families [78].

Although this phenomenon has been characterized primarily in human models, it suggests a potential drug interaction of relevance in production settings, considering the concomitant use of antiparasitic agents or therapeutic antibiotics. However, these interactions have not been evaluated in species of zootechnical interest, which constitutes an additional safety gap that should be addressed prior to widespread commercial implementation.

The sensory impact of animal-derived products constitutes a practical barrier that is often underestimated due to the characteristic bitterness associated with hop α- and β- acids. The available evidence is limited, though encouraging. In tilapia, a consumer panel did not detect any perceptible differences in fillet aroma, whether raw or cooked, at the highest dose evaluated (1230 mg/kg). In poultry, meat quality studies reported no organoleptic rejection; however, these evaluations relied primarily on instrumental measurements and lacked formal sensory validation. Currently, there is no data on the potential transfer of bitter notes or off-flavors to milk or eggs, matrices in which the consumer detection threshold could be considerably lower. Consequently, systematic sensory evaluation using trained panels and consumer studies, specific to each product type (meat, milk, and eggs), constitutes an indispensable preliminary step for eventual commercial implementation.

Finally, commercial viability depends critically on cost-effectiveness, an aspect that has not yet been rigorously quantified in the scientific literature. Hops present a significant structural advantage in this context, as a substantial fraction of the bioactive compounds can be obtained from byproducts and waste from the brewing industry, including spent hops, trub, and brewer’s yeast, potentially reducing the cost of raw materials compared to phytogenic additives cultivated specifically for this purpose. However, this economic potential may be partially offset by costs associated with analytical standardization, microencapsulation, or bitterness masking, as well as by seasonal variability in raw material supply. In this context, it is essential to develop formal techno-economic analyses that compare the cost per unit of productive effect, for example, cost per point of improvement in the feed conversion ratio, against antibiotic growth promoters and other phytogenic additives, while also incorporating formulation and regulatory compliance costs. The absence of such evaluations currently represents one of the main barriers to the transition of hops from proof-of-concept studies to effective commercial adoption.

### 5.6. Palatability, Feed Intake, and Bitterness Mitigation Strategies

The characteristic bitterness of hops, attributable mainly to α- and β-acids [1,3], constitutes one of the main practical barriers to their use as additives in animal feed. These compounds activate bitter taste receptors of the TAS2R family, whose stimulation can reduce voluntary feed intake when high inclusion levels are used. This interaction is not merely hypothetical in the case of hops. As discussed in Section 4.3, the improvement in feed conversion ratio observed in finishing pigs supplemented with granulated cones [64], was largely attributed to a significant reduction in voluntary intake. This finding illustrates the difficulty of distinguishing between genuine productive effects and responses induced by reduced palatability.

Sensitivity to bitterness shows marked interspecific variation, which modulates the expected magnitude of this effect. Birds possess a considerably smaller repertoire of TAS2R genes than mammals [79], with only three functional receptors described in chickens [80], in addition to a taste bud density substantially lower than that of pigs and humans [81]. This sensory architecture could explain the greater tolerance observed in poultry compared with other terrestrial monogastric animals, although the reduced receptor repertoire is partially compensated for by receptors with a broader tuning range [80]. In contrast, bitterness perception in farmed fish remains poorly characterized, limiting the extrapolation of acceptance thresholds between aquaculture and terrestrial systems. Despite its practical relevance, the available literature still lacks systematic palatability studies, including cafeteria-style preference tests and dose–response curves for voluntary feed intake, specific to each species and production category.

Among mitigation technologies, microencapsulation is one of the most promising strategies, as it enables masking of the bitter stimulus while simultaneously protecting bioactive compounds from degradation during processing and gastrointestinal transit. Applicable techniques include spray drying, coacervation, inclusion complex formation with cyclodextrins, and lipid-based or liposomal systems. Complementary strategies include the use of flavor-masking agents, gradual adaptation of animals to the additive, and the utilization of brewing byproducts with lower residual bitter acid content. However, the encapsulation efficiency of these matrices has not been validated under the actual pelleting and extrusion conditions typical of industrial feed manufacturing. Similarly, their effects on the bioavailability of target compounds and final production performance have not been systematically evaluated. The quantification of the economic impact of these technologies, discussed in Section 5.5, reinforces the need to address palatability together with commercial viability.

## 6. Conclusions

The reviewed evidence positions hop (*Humulus lupulus* L.) as a phytochemical resource with high translational potential for the development of more sustainable animal nutrition strategies. Its matrix of secondary metabolites, particularly α- and β-acids, prenylflavonoids, and phenolic compounds, exhibits biological activities relevant to current challenges in animal production, including reducing antibiotic use, controlling enteric and fungal pathogens, modulating intestinal and ruminal health, and improving quality attributes in animal-derived products.

The most robust in vivo evidence comes from poultry, where hop β-acids have demonstrated anticlostridial activity, modulation of the intestinal inflammatory response, and favorable effects on the oxidative quality and lipid profile of chicken meat under specific dosage and administration conditions. In freshwater aquaculture, particularly in common carp and Nile tilapia, the available studies remain scarce and heterogeneous, but provide a biologically plausible proof of concept for the use of bitter acids and hop extracts in antifungal control, hepatoprotection, and improvement of the fillet fatty acid profile. In swine, the evidence remains preliminary, although it suggests favorable effects on feed efficiency and meat composition during the finishing phase. In ruminants, in vivo studies indicate that dry or ensiled residues, pellets, and hop cones can be incorporated into sheep and cattle diets without significant adverse effects on intake, growth, or carcass characteristics. However, their productive benefits appear limited and seem to be primarily associated with the modulation of ruminal fermentation, nitrogen utilization, and energy metabolism, rather than with direct and consistent improvements in production efficiency.

Overall, the reviewed studies indicate responses that depend on dose, species, chemical form, and the administered matrix, underscoring the need for formulation and dosing strategies tailored to each production system. The commercial implementation of hops as a functional additive requires addressing critical gaps, including species-specific pharmacokinetic and metabolic characterization, validation of efficacy in challenge models with relevant pathogens, comprehensive evaluation of effects on the gastrointestinal microbiota, development of technologies to mitigate the palatability limitations associated with the bitterness of α- and β-acids, and preparation of regulatory dossiers addressing maximum residue limits and the potential transfer of prenylflavonoids with estrogenic activity to edible matrices. Only through the integration of mechanistic, productive, microbiological, and regulatory evidence will it be possible to move from phytochemical promise toward a safe, effective, and commercially viable application in animal nutrition.

## Figures and Tables

**Table 3:** Summary of in vivo and ex vivo studies evaluating the effects of *H. lupulus* supplementation on growth performance, intestinal health, immune modulation, and meat quality in broiler chickens (*Gallus gallus domesticus*).

Species/Model	Extract/Compound	Dose & Duration	Parameters Evaluated	Main Findings	Production Relevance	Reference
*Gallus gallus domesticus* (broiler)	Lupulone (β-acid), administered in drinking water	62.5, 125, and 250 ppm in drinking water; 9 days (days 13–22 post-hatch)	Intestinal *C. perfringens* counts (jejunum and caecum); body weight; liquid consumption	Lupulone reduced intestinal *C. perfringens* counts at day 22 in both jejunum and caecum across all doses, with no significant effect observed at day 17	Necrotic enteritis control—major economic disease in commercial poultry	[33]
*Gallus gallus domesticus* (broiler)	Lupulone (β-acid), administered in drinking water	125 mg/L in drinking water; 9 days (days 13–22 post-hatch)	Gut microbiota composition	↓ *Clostridium* spp. in cecum without affecting *Lactobacillus* spp. Selective prebiotic-like modulation—unique profile absent in conventional antibiotics.	Antibiotic replacement—selective pathogen reduction; however, concurrent reduction in beneficial *Lactobacillus* in midgut warrants further investigation	[34]
*Gallus gallus domesticus* (broiler)	Hops β-acids (microencapsulated), dietary inclusion	30, 60, 120, and 240 mg/kg in feed; 42 days (1–42 d)	Body weight, feed intake, FCR, jejunum villus morphology, *Clostridium* spp. counts (jejunum and cecum)	Hop β-acids improved FCR, with the strongest response at 30 mg/kg, while villus morphology was unaffected and *Clostridium* spp. were virtually absent across treatments.	Antibiotic replacement—β-acids at 30 mg/kg improved FCR equivalently to zinc bacitracin under intestinal challenge conditions	[35]
*Gallus gallus domesticus* (pullet, ex vivo intestinal tissue)	Hop β-acids (45% extract)	30 and 240 mg/kg; 1 h incubation (ex vivo model)	Intestinal gene expression of IL-1β, IL-6, IFN-γ, IL-4, and IL-10	↓ IL-1β at both doses; ↓ IFN-γ only at 240 mg/kg; 30 mf/kg counteracted LPS-induced IL-1β upregulation; no significant effect on IL-6, IL-4, or IL-10	Mucosal immune regulation in poultry under enteric challenge	[45]
*Gallus gallus domesticus* (broiler, Cobb 500)	Hop β-acids (microencapsulated)	0, 30, 60, and 240 mg/kg in feed; 42 days (1–42 d)	Meat polar metabolite profile, fatty acid composition, redox stability, myofibrillar protein oxidation	Hop β-acids, especially at 30 mg/kg, improved meat redox stability, enhanced antioxidant-related metabolites and PUFA content, and reduced myofibrillar protein oxidation	Meat quality improvement—dietary β-acids at 30 mg/kg enhance oxidative shelf life and nutritional value of broiler breast meat without sensory impact	[43]
*Gallus gallus domesticus* (Ross 308 male broilers)	Ground hop cones; blend of varieties 94/127 and 108/78 (50:50); rich in α-acids, β-acids and polyphenols	0, 0.9 and 3.6 g hops/kg feed; high-PUFA diet (7.5% linseed oil); 37 days	Growth performance, oxidative stress markers, antioxidant capacity, DNA damage, vitamin E status, and basic meat quality traits	High-dose hop cones reduced weight gain and increased lipid oxidation markers in plasma and fresh meat, while lowering γ-tocopherol; however, DNA fragmentation decreased and basic meat quality traits were largely unaffected.	High-dose hop cones reduced performance and increased oxidative instability in PUFA-rich diets; the low dose was safe but not antioxidant	[59]

FCR: feed conversion ratio; IFN-γ: interferon gamma; IL-1β: interleukin-1 beta; IL-4: interleukin-4; IL-6: interleukin-6; IL-10: interleukin-10; PUFA: polyunsaturated fatty acid; ↓: Indicates a decrease in the reported parameter.

## Data Availability

No new data were created or analyzed in this study. Data sharing is not applicable to this article.
